# A Novel Quantum Dot-Based pH Probe for Long-Term Fluorescence Lifetime Imaging Microscopy Experiments in Living Cells

**DOI:** 10.1021/acsami.1c19926

**Published:** 2022-01-10

**Authors:** Diego Herrera-Ochoa, Pedro J. Pacheco-Liñán, Iván Bravo, Andrés Garzón-Ruiz

**Affiliations:** † Departamento de Química Física, Facultad de Farmacia, Universidad de Castilla-La Mancha, Av. Dr. José María Sánchez Ibáñez, s/n, 02071 Albacete, Spain; ‡ Centro Regional de Investigaciones Biomédicas (CRIB), Unidad Asociada de Biomedicina (UCLM-CSIC), C/Almansa, 14, 02008 Albacete, Spain

**Keywords:** FLIM, fluorescence lifetime probe, intracellular pH quantification, histidine-based nanoparticle, quantum dot

## Abstract

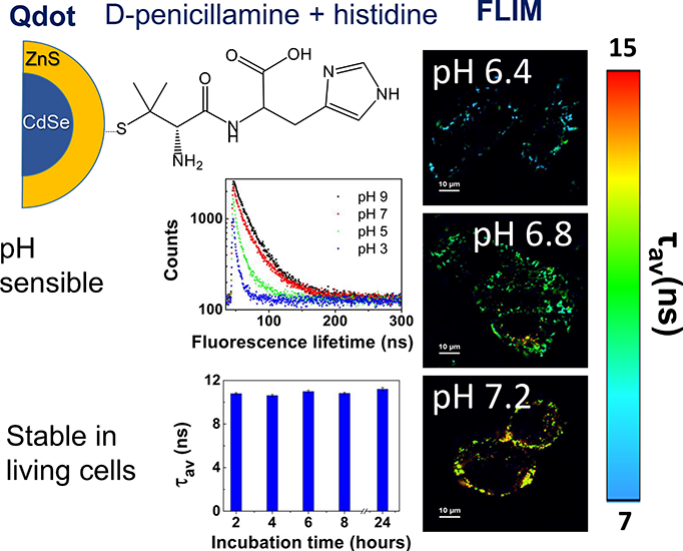

The use of two nanoparticles for quantitative pH measurements in live cells by means of fluorescence lifetime imaging microscopy (FLIM) is investigated here. These nanoparticles are based on CdSe/ZnS quantum dots (QDs), functionalized with *N*-acetylcysteine (CdSe/ZnS-*A*) and with a small peptide containing D-penicillamine and histidine (CdSe/ZnS-*PH*). CdSe/ZnS-*A* has tendency to aggregate and nonlinear pH sensitivity in a complex medium containing salts and macromolecules. On the contrary, CdSe/ZnS-*PH* shows chemical stability, low toxicity, efficient uptake in C3H10T1/2 cells, and good performance as an FLIM probe. CdSe/ZnS-*PH* also has key advantages over a recently reported probe based on a CdSe/ZnS QD functionalized with D-penicillamine (longer lifetimes and higher pH-sensitivity). A pH(±2σ) of 6.97 ± 0.14 was determined for C3H10T1/2 cells by FLIM employing this nanoprobe. In addition, the fluorescence lifetime signal remains nearly constant for C3H10T1/2 cells treated with CdSe/ZnS-*PH* for 24 h. These results show the promising applications of this nanoprobe to monitor the intracellular pH and cell state employing the FLIM technique.

Biomarkers are used to monitor metabolic and physiological changes at the cellular level caused by diseases, drugs, and toxic substances, among others. Out of all of them, pH is a useful biomarker of the cell state^1^ because of the presence of various cell mechanisms to maintain the intracellular pH around 7.2, although this valor may be different in certain organelles.^2^ Processes such as apoptosis, as well as the presence of exogenous compounds, such as drugs and toxic substances, can alter the intracellular pH.^3−5^ Therefore, the intracellular pH can also be an interesting biomarker for the monitoring of cancer and other degenerative diseases.^6−8^

Recently, we showed the potential of fluorescence lifetime imaging microscopy (FLIM) for pH quantification in living cells.^9^ FLIM has significant advantages for quantitative measurements compared to conventional fluorescence intensity microscopy; that is, the fluorescence lifetime is not sensitive to either the fluorophore concentration, excitation source intensity, or duration of light exposure.^10−12^ In addition, the effect of cellular autofluorescence lifetime (1–2 ns) can be minimized or eliminated by the use of long-lifetime fluorescence probes.^9,11,12^

Few examples of quantitative intracellular pH measurements by FLIM in live cells, employing fluorescent nanoparticle probes, have been reported.^9,13−15^ Different functionalized CdSe/ZnS core–shell quantum dots (QDs) and carbon dots (CDs), as well as a perylene bisimide derivative encapsulated in a nanopolymer, were used as pH probes in those studies.^9,13,14^ In general, larger lifetimes have been reported for QD-based nanoprobes, in comparison to those based on a CD or nanopolymer.^9,13−15^ An L-glutathione functionalized QD has also been used as a pH nanoprobe for in vivo imaging.^16^

The CdSe/ZnS QD (CdSe/ZnS-*P*) functionalized with D-penicillamine was successfully used as an intracellular FLIM pH probe in our laboratory. This nanoparticle exhibited stability against aggregation, low cytotoxicity, easy cell penetration, and a linear relationship between fluorescence lifetime and pH in the physiological range.^9^ Unfortunately, the stability problems of the fluorescence lifetime in the cell medium in long-term experiments (several hours) limited the use of this nanoprobe. In this context, there is a need for novel nanoparticles with improved photophysical properties that allow monitoring pH in live cells during long-term experiments. Such properties are strongly influenced by the type of ligand employed for the nanoparticle functionalization. For instance, the microenvironment hydrophobicity and protection against fluorescence quenching are two key factors determined by the ligand. In addition, the presence of ionizable groups increases the solubility of the nanoparticle and provides pH sensitivity.

In the present work, two new nanoparticles have been synthetized by functionalization of a CdSe/ZnS QD. In the first one, *N*-acetylcysteine was used as a ligand because of its structural similarity to D-penicillamine, the absence of methyl groups near the thiol group (the linker to the QD surface), and the presence of an amide group (CdSe/ZnS-*A*). Second, a peptide containing D-penicillamine and histidine (CdSe/ZnS-*PH*) was employed as a ligand in the search for a more hydrophobic microenvironment around the QD (see Figure 1). In this sense, partition coefficients at pH 7.4 (log *D*^7.4^) of 0.026 and 0.47 have been reported for related dipeptides containing histidine such as L-carnosine (β-alanyl-L-histidine) and L-Cys-L-His-OMe.^17,18^ These dipeptides are more hydrophobic than single amino acids such D-penicillamine (log *D*^7.0^ = −1,78) and histidine (log *D*^7.0^ = −3.56). Both CdSe/ZnS-*PH* and CdSe/ZnS-*A* were tested as pH nanoprobes for living cells, emphasizing stability studies of the fluorescence lifetime with CdSe/ZnS-*PH* in C3H/10T1/2 cells for 24 h. This is a key point for the potential use of this probe to monitor the effect of drugs, toxins, and other substances on cells.

**Figure 1:**
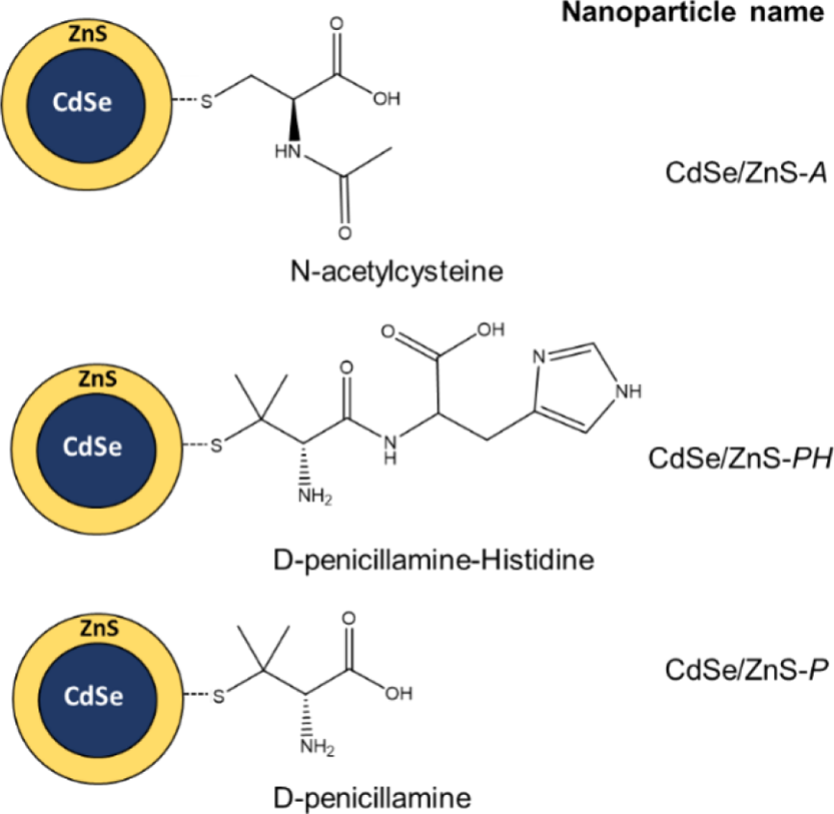
Chemical representation of the studied nanoparticles.

## Experimental Section

A detailed description of the employed reagents and methodologies can be found in the Supporting Information. Nevertheless, a summary with the most relevant information is shown as follows:

### QD Functionalization

The functionalization of CdSe/ZnS-*PH* and CdSe/ZnS-*A* was based on the studies reported by Pratiwi et al.^19^ and Chen et al.,^20^ respectively.

### Nanoparticle Characterization

The nanoparticles were characterized by dynamic light scattering (DLS) and spectroscopic measurements as well as transmission electron microscopy (TEM) images. Ligand grafting densities were estimated by UV–vis absorption spectroscopy measurements.

### pH-Sensitivity: Spectroscopic Studies

The dependence of the fluorescence emission intensity and lifetime with pH was studied in a FLS920 spectrofluorometer (Edinburgh Instruments). In time-resolved experiments, the excitation and emission wavelengths were fixed at 565 and 626 nm, respectively. The temperature of the sample was fixed at 20 °C. These experiments were performed in both Tris–HCl buffer solutions and a synthetic intracellular buffer (SIB) that mimics the intracellular environment. The nanoparticle concentration was 10 nM.

The fluorescence decay profiles were fitted by the following multiexponential function:
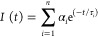
1α_*i*_ being the amplitude and τ_*i*_ and fluorescence lifetime for each *i*^th^ term. An average decay lifetime (τ_av_) was obtained through the following equation:
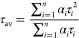
2

The experiment was repeated at least three times for each selected pH value.

### Cell Cultures and Viability Assays

*Mus musculus* embryo fibroblasts (C3H10T1/2; ATCC CCL-226) were cultured as described in ref (9). MTT assays were performed to assess the cytotoxicity of the functionalized nanoparticles on C3H/10T1/2 cells (see ref.^9^ for details).

### Fluorescence Lifetime Imaging in Cells

C3H/10T1/2 cells were seeded onto 20 mm square glass cover slides and incubated with the functionalized nanoparticles (50 nM) for 60 min, as previously described in ref (9). After the treatment, FLIM images of the cells were acquired using a MicroTime 200 microscope (PicoQuant). Samples were excited at 511 nm with a diode pulsed laser. In each FLIM image, a region of 80 × 80 μm was scanned with a spatial resolution of 156 nm/pixel. In each pixel, the fluorescence intensity decay was fitted to eq 1, and the average decay lifetime was calculated following eq 2. The average fluorescence lifetime of the whole FLIM image was obtained from the maximum of the best fitting Gaussian curve of the lifetime distribution histogram. At least, three independent cell samples (and six FLIM images per sample) were measured. The intracellular pH of C3H/10T1/2 cells was artificially modified to test the pH-sensitivity of CdSe/ZnS-*PH* employing a previously described procedure.^9^

## Results and Discussion

### Nanoparticle Functionalization and Characterization

Different ligand/QD ratios were employed in the functionalization of CdSe/ZnS-*PH* and CdSe/ZnS-*A* nanoparticles. It is well known that the linkage between CdSe/ZnS and thiolated compounds is produced by Zn-S covalent bonds.^9,21−23^ The fluorescence emission of nanoparticles increased as a function of the ligand/QD ratio because ligands improve the water solubility and reduce the quenching (see Figure S1). Plateau values were reached for ratios 5000:1 and 7000:1 for CdSe/ZnS-*PH* and CdSe/ZnS-*A*, respectively. Under such ratios, we considered that the number of bound ligands reached the maximum. Table 1 shows the number of ligands per unit area on the QD surface (see Table S1 and the Experimental Section for additional details). Nanoparticle sizes (diameter) of 8.1 ± 0.6 and 13.7 ± 1.1 nm were measured for nonfunctionalized CdSe/ZnS and CdSe/ZnS-*PH*, respectively, by TEM (see Figures S7 and S8). The ligand grafting densities obtained for the functionalized nanoparticles (3.3–8.9 ligands nm^–2^) are within the order of magnitude of other values reported for CdSe, PbSe, and PbS QDs (0.6–11 ligands nm^–2^).^24^ The lowest grafting density was found for CdSe/ZnS-*PH* in agreement with its higher ligand size (as mentioned above, a lower ligand/QD ratio was also used in the functionalization of this nanoparticle). A smaller number of ligands per nm^2^ were estimated for CdSe/ZnS-*A* when compared to the reference nanoparticle (CdSe/ZnS-*P*). Therefore, each molecule of *N*-acetylcysteine occupies a higher surface on the QD than a D-penicillamine ligand. This fact could be related to the molecular branching of *N*-acetylcysteine at the C_β_ position with respect to the Zn-S linkage.

**Table 1:** Size (Hydrodynamic Radius), Zeta (ζ) Potential, and Fluorescence Quantum Yield (Φ_F_) of the Studied Nanoparticles at Different pH Values

property	pH	CdSe/ZnS-*PH*	CdSe/ZnS-*A*	pH	CdSe/ZnS-*P*^a^
r. size (nm ± 2σ) [PdI]^b^	3.0	600 ± 97 [0.40]	–c	–	>450
	5.0	24.5 ± 2.0 [0.23]	12.9 ± 1.8 [0.29]	–	–
	7.0	10.8 ± 1.0 [0.21]	8.4 ± 0.8 [0.08]	7.2	13.7 ± 6.1 [0.49]
	9.0	10.6 ± 1.6 [0.24]	13.4 ± 1.4 [0.38]	9.0	12.7 ± 2.7 [0.52]
ζ-potential (mV ± 2σ)^b^	3.0	–34.2 ± 1.8	–c	4.0	–25.2 ± 2.3
	5.0	–48.5 ± 2.1	–12.4 ± 0.3	–	–
	7.0	–55.5 ± 1.8	–16.5 ± 2.4	7.2	–27.8 ± 5.9
	9.0	–58.6 ± 1.7	–27.0 ± 0.9	9.0	–40.2 ± 1.4
number of ligands/nm^2^	7.0	3.3	6.4	7.0	8.9
Φ_F_ (%)	9.0	32	35	9.0	22

a Ref (9).

b The work concentration was 0.6 μM.

c Nanoparticle aggregation.

DLS experiments showed that CdSe/ZnS-*PH* and CdSe/ZnS-*A* have comparable sizes which significantly increase at pH = 3, probably because of aggregation phenomena (see Table 1 and Figures S2 and S3).^9,25,26^ On the contrary, small changes in the nanoparticle size were observed within pH 5–9 and, therefore, no significant aggregation is expected at physiological pH (at least during several hours). As previously reported for CdSe/ZnS-*P*,^9^ the zeta potentials indicate that a negatively charged shell gradually appears on the nanoparticle surface when pH increases because of the deprotonation of the carboxylic groups. The zeta potential is at least two times higher for CdSe/ZnS-*PH* than for CdSe/ZnS-*A*, independent of pH, suggesting that the first nanoparticle is more stable against aggregation. In a further experiment, it was observed that the particle size of CdSe/ZnS-*PH* remains almost unaltered for at least 3 days at pH 7.4 (Figure S4), while precipitation was observed for CdSe/ZnS-*A* in the first 24 h.

Both CdSe/ZnS-*PH* and CdSe/ZnS-*A* present similar spectral profiles to CdSe/ZnS-*P* with characteristic broad absorption and excitation bands and a narrow emission peak centered at 627 nm (see Figure S5).^9^ It is worth noting that the increase of the fluorescence lifetime of these nanoparticles with respect to CdSe/ZnS-*P* is a key improvement for FLIM applications, that is, τ_av_ ∼ 25 ns for CdSe/ZnS-*PH* (pH 7–9), τ_av_ ∼ 18 ns for CdSe/ZnS-*A* (pH 6–8), and τ_av_ ∼ 14 ns for CdSe/ZnS-*P* (pH 8–9). It was also found that the longer length of -*PH* peptides compared to D-penicillamine leads to higher protection against fluorescence quenching. Employing glucose as a quencher, a Stern–Volmer constant (*K*_SV_) about two times higher was determined for CdSe/ZnS-*P* (2.9 × 10^5^ M^–1^) than for the peptide-functionalized nanoparticle (1.7 × 10^5^ M^–1^) (see Figure S9). Higher fluorescence quantum yields were also determined for both CdSe/ZnS-*PH* and CdSe/ZnS-*A* with respect to the value reported for CdSe/ZnS-*P* (see Table 1).

### pH-Sensitivity

**Figure 2:**
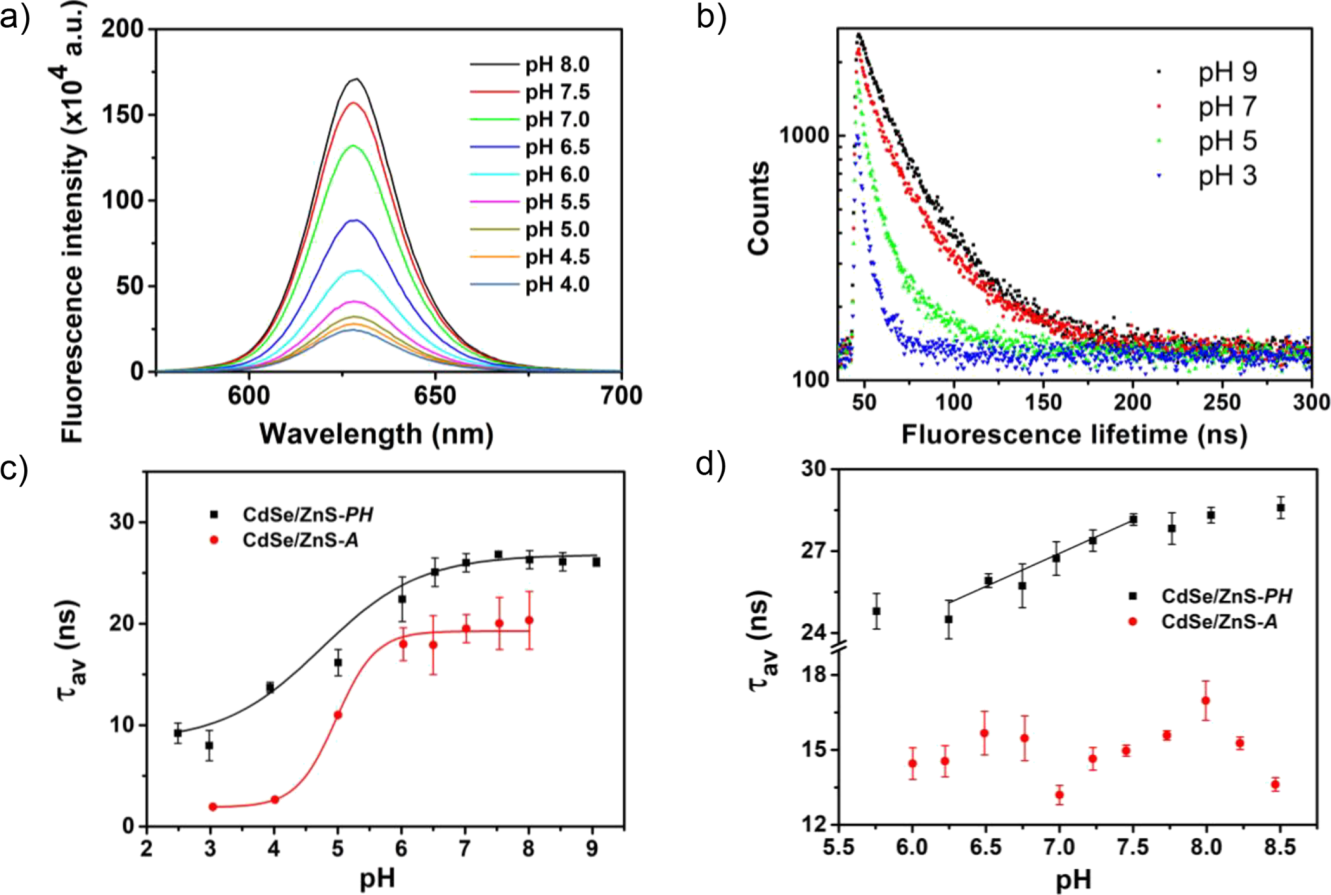
Fluorescence pH-sensitivity: (a) Fluorescence emission spectra recorded for CdSe/ZnS-*PH* in Tris–HCl buffer solution. (b) Fluorescence emission decay profiles acquired for CdSe/ZnS-*PH* in Tris–HCl buffer solution. Dependence of τ_av_(±σ) with pH obtained for CdSe/ZnS-*PH* and CdSe/ZnS-*A* in (c) Tris–HCl buffer solution and (d) SIB solution (the solid lines correspond to the sigmoid and linear fits). The nanoparticle concentration was 10 nM in all these experiments.

Figures 2a and S6a show the dependence of the fluorescence emission intensity as a function of pH. The fluorescence intensity of both CdSe/ZnS-*PH* and CdSe/ZnS-*A* increases with pH without a significant spectral shift as previously reported for CdSe/ZnS-*P.*^9^ Both nanoparticles exhibited multiexponential fluorescence decay profiles which are also sensitive to the pH of the medium (see Figures 2b and S6b). In addition, the pH-sensitive response is reversible between pH 6.5 and 7.5 for both nanoparticles (see Figure S10).

Figure 2c shows τ_av_ vs pH-titration curves obtained for the studied nanoparticles in aqueous solution (see Table S2 for details). Both curves were fitted by the following sigmoidal equation
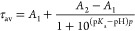
3where τ_av_ is the fluorescence lifetime, *A*_1_ and *A*_2_ are the bottom and top asymptotes, and *p* is the Hill slope. The fitting parameters are shown in Table S3. A p*K*_a_ value of 5.0 was determined for CdSe/ZnS-*A* which has only a single ionizable group per ligand molecule. On the contrary, lower p*K*_a_ values (3.0–3.1) have been reported for the first protonation equilibrium (ionization of the carboxyl group) of free *N*-acetylcysteine in aqueous solution.^27−29^ This p*K*_a_ shift of *N*-acetylcysteine upon binding to the QD can be attributed to the more hydrophobic environment of the nanoparticle along with cooperativity effects between molecules (i.e., the carboxyl groups initially ionized could stabilize the acid proton of the nearby neutral carboxyl groups, making it more difficult to remove by the free hydroxide ions in the bulk solution).^30,31^ Similarly, shifts of p*K*_a_ toward higher values have been observed in fatty acids after chain elongation because of a more hydrophobic environment and greater interactions among carboxyl groups.^30,31^

As discussed above, p*K*_a_ and zeta potential values determined for CdSe/ZnS-*PH* suggest that pH-sensitivity within the range 3–7 is governed by the deprotonation of the primary amine group in concordance with the reference nanoparticle, CdSe/ZnS-*P*. p*K*_a,*i*_ values of 2.57, 6.71, and 9.57 have been reported for carnosine, a dipeptide structurally related to *PH.*(32) Starting from the fully protonated form of carnosine, p*K*_a,1_ and p*K*_a,2_ correspond to the dissociation equilibria of the carboxyl group and 1H-imidazole ring, respectively (both located in the C-terminal residue, i.e., L-histidine). 1H-imidazole rings of CdSe/ZnS-*PH* may be in the nonionized form around pH 7 because this nanoparticle has a higher (negative) zeta potential than CdSe/ZnS-*P* even with a lower ligand grafting density. Thus, the pH-titration curve obtained for CdSe/ZnS-*PH* would be associated with the dissociation equilibrium of the N-terminal amine group. The p*K*_a_ determined for CdSe/ZnS-*PH* (4.7) is significantly lower than the p*K*_a,3_ reported for carnosine^32^ and the p*K*_a,2_ reported for the dissociation equilibrium of the primary amine group of free D-penicillamine (7.9).^33^ This downward shift in p*K*_a_ (as well as the previously discussed upward shift in p*K*_a_ observed for CdSe/ZnS-*A*) may be associated with the improved stability of the neutral forms of the ionizing groups inside the hydrophobic environment of the nanoparticle ligand shell and the “cooperativity” effects, as commented above. A p*K*_a_ shift to lower values was also reported for CdSe/ZnS-*P* (5.7) when compared to free D-penicillamine. The lower p*K*_a_ determined for CdSe/ZnS-*PH* than for CdSe/ZnS-*P* agrees with the higher zeta potentials measured for the former nanoparticle at pH 7 (a higher population of primary amines should be in the neutral state for CdSe/ZnS-*PH* than for CdSe/ZnS-*P*, increasing the negative charge of the former nanoparticle at pH 7).

According to the previous discussion, it seems that the protonation of the -NH_2_ groups of CdSe/ZnS-*PH* is the main factor associated with the decrease of the fluorescence intensity and lifetime observed upon acidification of the medium (see Figure 2a–c). As density functional theory showed for CdSe/ZnS-*P*, the amine group of D-penicillamine interacts more strongly with the sulfur atoms of the ZnS shell in the ionized state than in the neutral form.^9^ Positive ions onto the QD surface can attract the photogenerated electron and repel the hole, increasing the spatial separation between both charge carriers.^34^ This mechanism would increase the energy-relaxation channels and decreases the rate of the radiative transition from the ground-state exciton state to the vacuum state leading to shorter fluorescence lifetimes and lower fluorescence intensities.^34^ This phenomenon has also been observed and studied in mercaptopropionic acid-functionalized CdSe-CdS/ZnS QDs in the presence of Ca^2+^ ions.^21^ The case of CdSe/ZnS-*A* is different because its ligands only have an ionizable group. Fluorescence self-quenching associated with aggregation processes could be related to the pH-sensibility of the nanoparticle. In fact, the lowest zeta potentials were obtained for this nanoparticle within the pH range of 5–9 with precipitation observed in the first 24 h.

The pH-sensitivity range found for CdSe/ZnS-*PH* (from pH ∼3 to 7) is significantly broader than that reported for CdSe/ZnS-*P* (from pH ∼5 to 7)^9^ while its Hill slope CdSe/ZnS-*PH* (*p* = 0.6) is lower than that found for the reference nanoparticle (*p* = 1.3).^9^ The length of the ligand chain also has an important effect on the bottom and top of the asymptote. Thus, the longer length of -*PH* peptides as compared to D-penicillamine increases the microenvironment hydrophobicity and protection against fluorescence quenching. Accordingly, it was observed that A_2_ increases from 14 ns for CdSe/ZnS-*P* to 27 ns for CdSe/ZnS-*PH*, while A_1_ varies from 4 ns for the reference nanoparticle to 8 ns for the *PH*-functionalized QD.^9^ As stated above, these high lifetime values are an important advantage for the use of CdSe/ZnS-*PH* in quantitative FLIM measurements.

The dependence of τ_av_ vs pH was also studied in a SIB that contains common cellular compounds such as ions, proteins, or polysaccharides (see Figure 2d). It is well known that the fluorescence of a nanoparticle can be affected by diverse physiological factors such as the concentration of macromolecules, ionic strength, and viscosity, among others.^35^ Significant differences were found between the pH-titration experiments carried out in SIB and the previous experiments performed in Tris–HCl buffered solutions. Whereas a wide linear response range was obtained for CdSe/ZnS-*PH*, which includes the physiological pH, a nonlinear response curve was found for CdSe/ZnS-*A* in SIB solution. In addition, as shown in Table 2, the pH sensitivity of CdSe/ZnS-*PH* (2.4 ns per pH unit) is higher than that reported for the reference nanoparticle (2.1 ns per pH unit).^9^ Therefore, CdSe/ZnS-*PH* seems to be a suitable candidate for quantitative measurements in live cells with FLIM: long fluorescence lifetime and high pH sensitivity in a medium that mimics the intracellular environment.

**Table 2:** Linear Fitting Parameters of τ_av_ vs pH Obtained for CdSe/ZnS-*PH* in SIB Solution and C3H10T1/2 Cells^a^

compound	medium	pH range	slope (*b* ± 2σ)	*Y*-intercept (*a* ± 2σ)	*r*^2^
CdSe/ZnS-*PH*	SIB	6.3–7.5	2.42 ± 0.21	9.98 ± 1.46	0.96
	C3H/10T1/2	6.4–7.2	2.04 ± 0.53	–3.39 ± 3.74	0.99
CdSe/ZnS-*P*^b^	SIB	6.0–9.0	2.05 ± 0.29	–2.95 ± 2.15	0.98
	C3H10T1/2	6.0–8.0	1.47 ± 0.06	–5.69 ± 0.46	0.99

a The fitting parameters of the reference nanoparticle, CdSe/ZnS-*P*, are also included for comparative purposes.

b Ref (9).

### Cytotoxicity and Cellular Uptake

**Figure 3:**
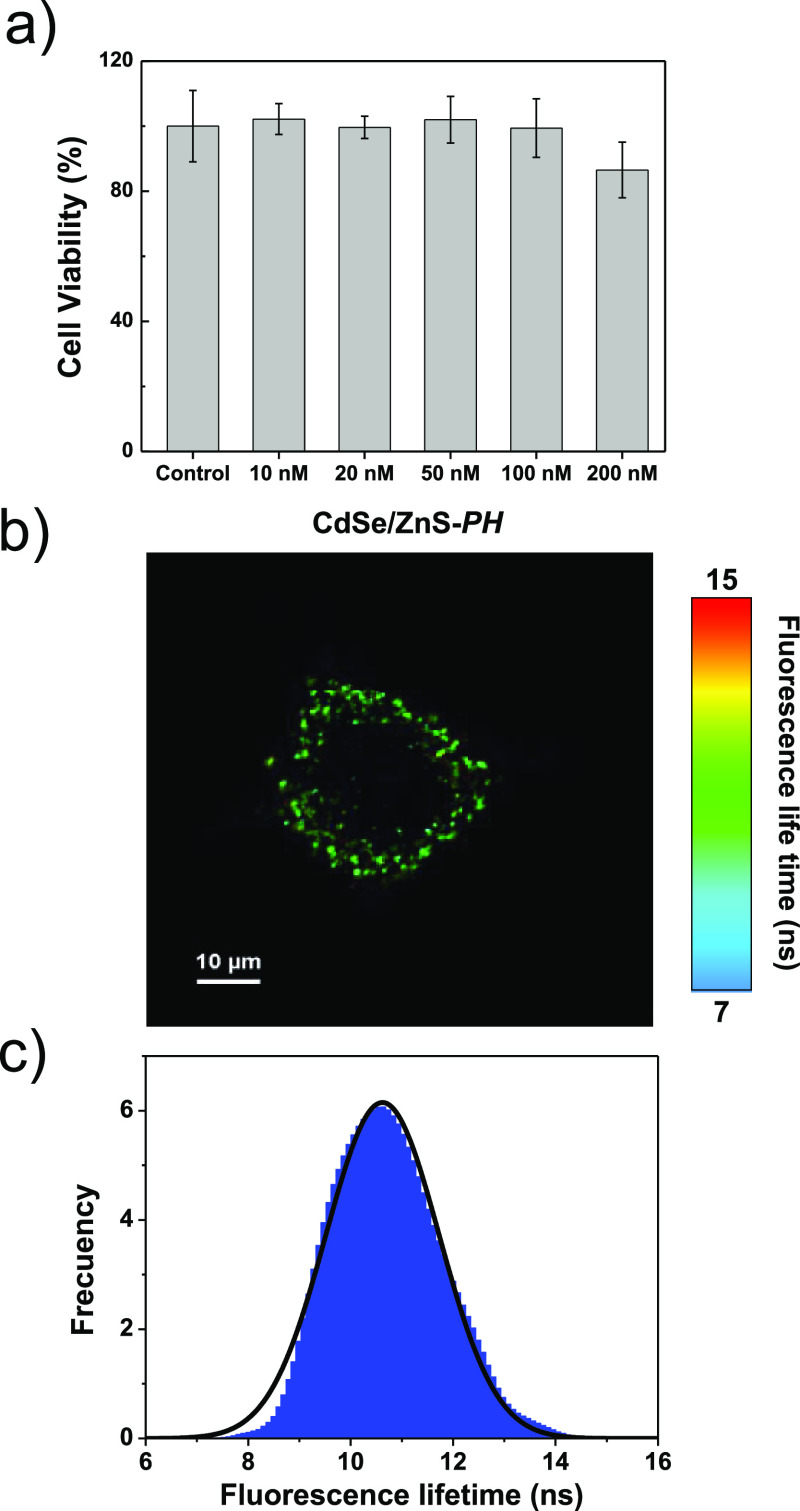
(a) Cytotoxicity of CdSe/ZnS-*PH* in C3H10T1/2 cells treated for 24 h at 37 °C. The control samples are C3H10T1/2 cells not treated with the nanoparticle (** *p* < 0.01 compared to the control group). (b) FLIM images of C3H10T1/2 cells treated with CdSe/ZnS-*PH* (50 nM) for 30 min. (c) Histogram of frequency vs lifetime corresponding to (b).

The cytotoxicity of CdSe/ZnS-*PH* and CdSe/ZnS-*A* was tested in C3H10T1/2 cells by MTT assay. Although QDs have been traditionally associated with toxic effects, other authors maintain that each nanoparticle exhibits different uptake kinetics, biodistribution patterns, degradation rates, and toxicity.^36^ The nanoparticle concentration, aggregation state, and chemical nature of ligands can play a fundamental role in their behavior in living systems. In this sense, Breus et al. suggested that the cytotoxicity of QDs can be reduced by functionalization with D-penicillamine, leading to biocompatible materials.^37^ In the present study, C3H10T1/2 cells were treated with the studied nanoparticles for 2 and 24 h. No significant effects on cell viability were found at 2 h of treatment (see Figure S11). For the 24-hour treatment, cell viability is affected by the presence of CdSe/ZnS-*PH* only in the upper range of tested concentrations (200 nM) (see Figure 3). Here, it should be remembered that the work concentration used in the FLIM experiments of the present work was 50 nM. Similar findings were reported for CdSe/ZnS-*P* and a D-penicillamine-functionalized Mn^2+^-doped QD.^9,19^ Over 90% of C3H10T1/2 cells remained viable after 24 h of treatment with CdSe/ZnS-*P* 200 nM.^9^ Similarly, the HeLa cell survival was higher than 90% after 48 h of treatment with the Mn^2+^-doped QD 325 nM.^19^ On the contrary, the cell viability is 50% or lower after a 24 h treatment with CdSe/ZnS-*A* for the whole tested concentration range. The higher cytotoxicity of this nanoparticle could be related to its lower chemical stability and aggregation tendency. Breus et al. reported that the steric effects of the methyl groups on the β-carbon of D-penicillamine reduce the interactions between ligands avoiding aggregation phenomena.^37^ Nevertheless, aggregation induced by oxidative dimerization of thiol ligands was observed in cysteine-functionalized QDs.^38^ As previously commented, we also observed precipitation for CdSe/ZnS-*A* at pH 7.2 in the first 24 h after solution preparation.

FLIM images show that CdSe/ZnS-*PH* was efficiently taken up into C3H10T1/2 cells and distributed in the cytoplasm (see Figures 3b and S12a). On the contrary, CdSe/ZnS-*A* did not efficiently enter C3H10T1/2 cells, and accumulation of this probe on the membrane was observed after 1 h of treatment (see Figure S13a). Weak fluorescence was observed for control cells in comparison with cells treated with the nanoparticles (see Figures S12c and S13c). The reduced effect of the autofluorescence can be attributed to the low laser power employed in the acquisition of FLIM images together with a large difference of ∼120 nm between the wavelengths of the excitation beam and the fluorescence emission.^9^ The value of τ_av_(±2σ) determined for C3H10T1/2 cells using CdSe/ZnS-*PH* as the probe was 10.84 ± 0.30 ns. Figure 3c shows the nearly Gaussian profile of the lifetime distribution histogram corresponding to Figure 3b and the fitting curve employed to obtain τ_av_. It is worth mentioning that this value of τ_av_ is significantly higher than the fluorescence lifetime measured in the same type of cell using CdSe/ZnS-*P* as the probe (4.50 ± 0.28 ns).^9^ Nevertheless, the fluorescence lifetime of CdSe/ZnS-*PH* in live cells is significantly lower than that in SIB solution at pH 7.0 (τ_av_ = 26.73 ± 1.22 ns). In this sense, it has been reported that the absorption of biomolecules (mainly proteins) on the nanoparticle surface and the acidic degradation associated with the action of lysosomes lead to a reduction of the fluorescence lifetime of homologous nanoparticles.^39^

### Cell pH-Sensing

**Figure 4:**
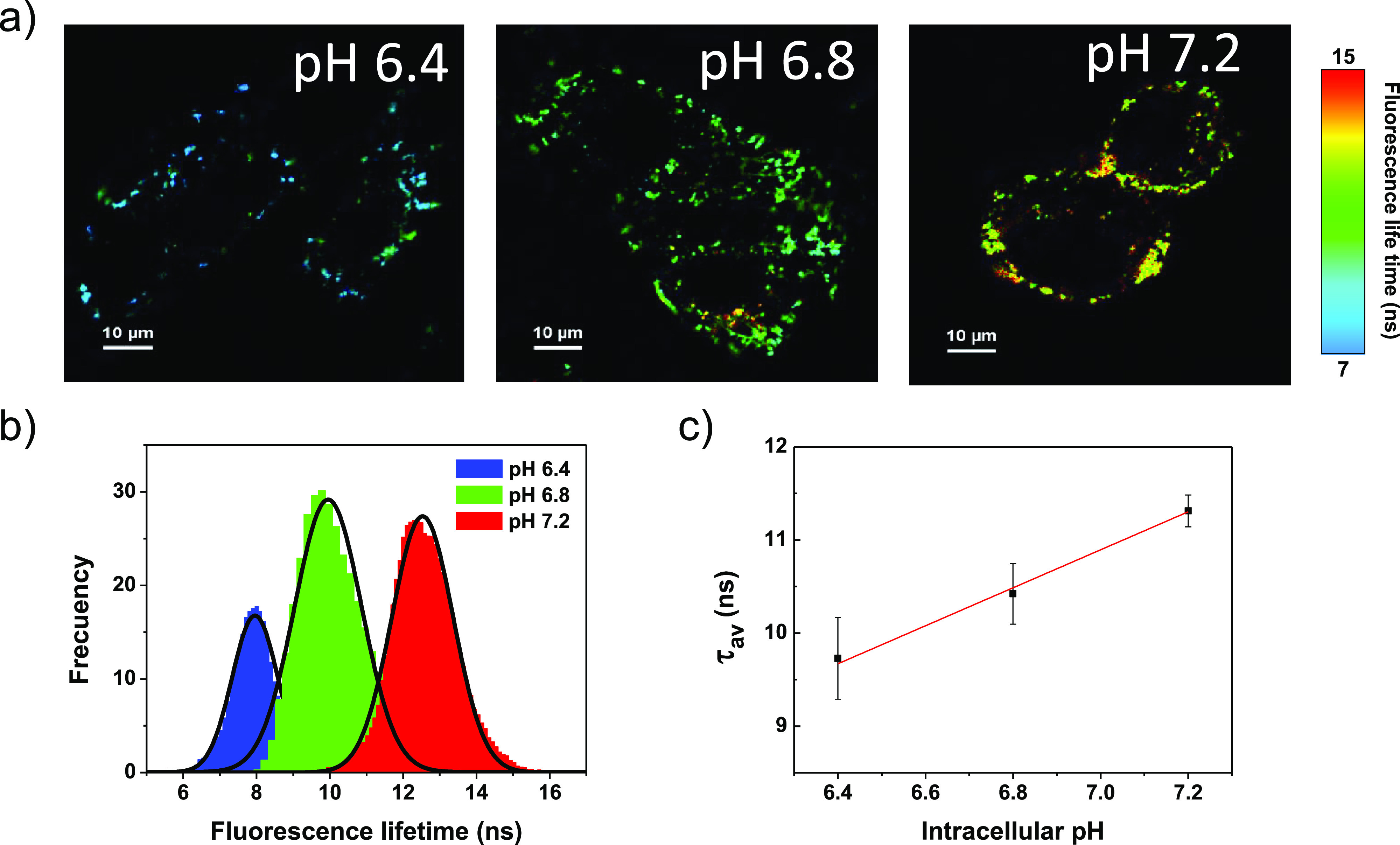
(a) FLIM images of C3H10T1/2 cells whose intracellular pH was artificially modified (cells were treated with CdSe/ZnS-*PH* (50 nM) for 30 min). (b) Fluorescence lifetime distribution histograms corresponding to the FLIM images of (a). (c) Linear fitting of τ_av_(±σ) vs pH.

The pH sensitivity in live cells was only assayed for CdSe/ZnS-*PH*. CdSe/ZnS-*A* was discarded because of the different drawbacks found, that is, aggregation tendency, toxicity, nonlinear pH-response in SIB, and inefficient uptake by cells. The FLIM images of Figures 4a and S14 illustrate the pH-sensitivity of the selected probe in C3H10T1/2 cells whose intracellular pH was artificially modified. The color scale of the FLIM images was arbitrarily chosen to allow an easy visualization of the changes in the fluorescence lifetime upon pH, that is, from blue color at pH 6.4 to greenish-yellow color at pH 7.2. Figure 4b shows the displacement of the lifetime distribution histogram upon the pH. A linear response range between pH 6.4 and 7.2 was found as pointed out in Figure 4c. The pH sensitivity of CdSe/ZnS-*PH* (2.0 ns per pH unit) is higher than that reported for the reference nanoparticle (1.5 ns per pH unit) (see Table 2).^9^ This feature and its long fluorescence lifetimes are key advantages of CdSe/ZnS-*PH* over the previously developed nanoprobe CdSe/ZnS-*P*. As reported for that nanoprobe, the τ_av_ vs pH calibration line obtained for CdSe/ZnS-*PH* is probably also dependent on the cell type and, therefore, a specific calibration line should be performed to obtain quantitative information in a different cell line.^9^ A pH(±2σ) value of 6.97 ± 0.14 was calculated for the intracellular medium of live C3H10T1/2 cells using the linear relationship of τ_av_ vs pH shown in Figure 4c. This result allows a cross-validation of the performance of both CdSe/ZnS-*PH* and CdSe/ZnS-*P* as pH probes for FLIM. In our previous work, we obtained a similar result for live C3H10T1/2 cells employing CdSe/ZnS-*P* as the probe (pH = 7.0).^9^ Comparable pH values have been reported for other mice fibroblast cells such as NIH/3 T3 (7.1–7.2)^40^ and L929 (7.0).^41^

Charged nanoparticles are generally introduced into the cell via endocytosis and transferred to lysosomes.^42^ Nevertheless, compounds with multiple amine groups can escape from endosomes to the cytosol according to the proton sponge hypothesis.^43^ The buffering capacity induces osmotic rupture in endosomes prior to fusion with lysosomes. The original hypothesis was proposed for polyethylenimine derivatives,^43^ but it has also been used to explain the escape capacity from endosomes of PAMAM (polyamidoamine) dendrimers,^44^ polyamine-DNA polyplexes,^45^ and QDs conjugated with PAMAM^46^ or D-glucosamine.^47^ We demonstrate herein that CdSe/ZnS-*PH* is sensitive to the artificial modification of the pH in the cell and, hence, it is not isolated in a cellular compartment. Similar findings were reported in previous FLIM studies on pH-sensitive QDs.^9,13^ In addition, we have not observed significant differences in the fluorescence lifetime histograms registered for the inner cytosol and more peripheral regions (see Figure S16). The presence of histidine residues in CdSe/ZnS-*PH* could help the endosomal escape. Some efficient gene delivery vectors based on polypeptide/DNA complexes containing histidine residues have been reported.^48,49^ It was proposed that the protonation of the imidazole groups in the endosome can favor the delivery of DNA into the cytosol.^49^

### Nanoparticle Stability in the Cell

**Figure 5:**
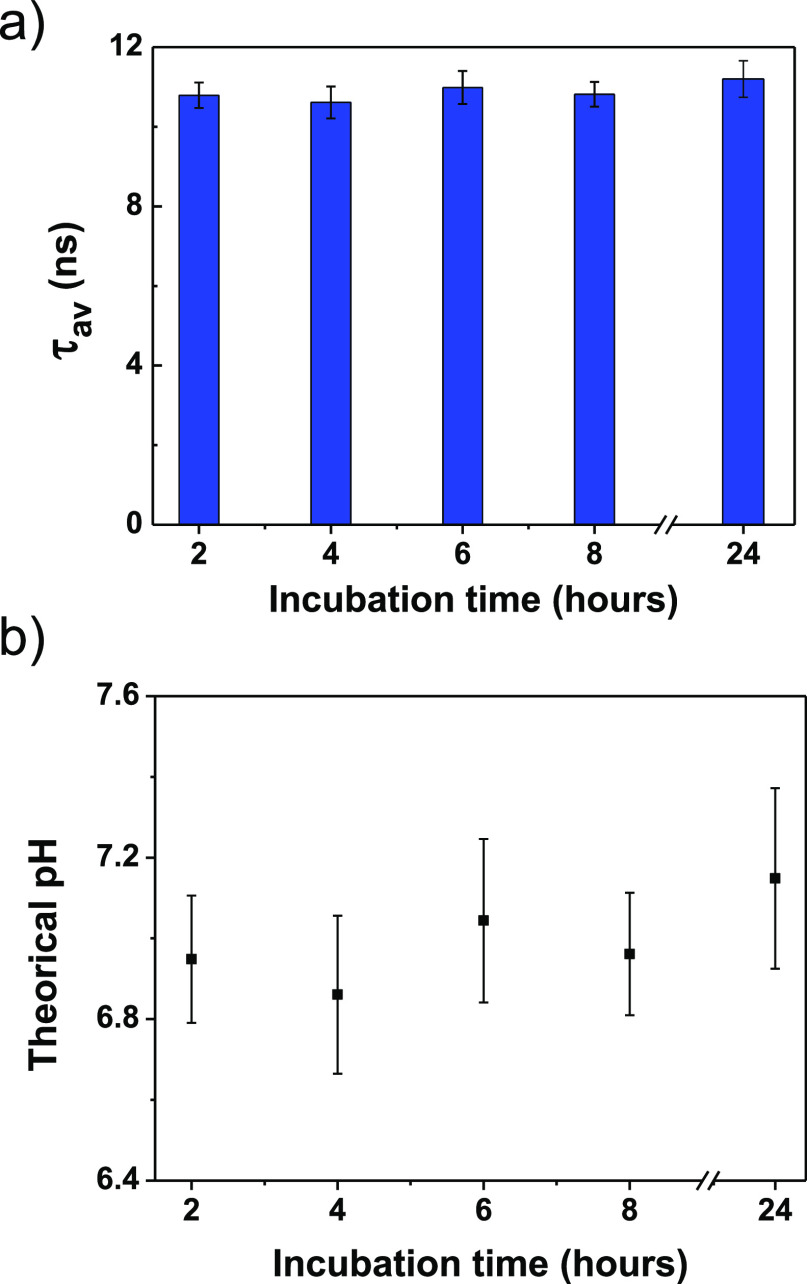
(a) Evolution of τ_av_(±σ) determined with FLIM for C3H10T1/2 cells treated with CdSe/ZnS-*PH* (50 nM) during different incubation times. (b) Estimated pH(±σ) for C3H10T1/2 cells treated with the FLIM probe during different incubation times.

The temporal stability of the fluorescence lifetime in complex media is a desired feature for FLIM probes with biological and biomedical applications. We found that the fluorescence lifetime of CdSe/ZnS-*PH* remains nearly constant in live C3H10T1/2 cells for 24 h (see Figure 5a). The lifetime distribution histograms obtained at different times also maintain a Gaussian profile (see Figure S15). All the intracellular pH values calculated in the 24-hour experiment are within the 6.9–7.1 pH range as shown in Figure 5b (assuming that the linear dependence of τ_av_ vs pH is maintained at different times). These results illustrate the promising applications of the probe CdSe/ZnS-*PH* in fluorescence lifetime bioimaging. The effect of diverse biomolecules, drugs, and toxic substances on the cell state can be monitored by pH measurements with FLIM and a suitable probe.

## Conclusions

A new nanoparticle (CdSe/ZnS-*PH*) composed of a CdSe/ZnS core–shell QD and D-penicillamine-histidine peptide as a ligand was developed. This nanoparticle exhibited chemical stability, pH sensitivity in the physiological range, low toxicity, and efficient uptake in C3H10T1/2 cells. Another nanoparticle (CdSe/ZnS-*A*) based on a core of CdSe/ZnS and *N*-acetylcysteine ligands was also synthesized, but it showed a tendency to aggregate and low pH sensitivity in a complex medium containing salts and macromolecules.

CdSe/ZnS-*PH* exhibited a good performance as the FLIM probe for quantitative intracellular pH measurements. In addition, CdSe/ZnS-*PH* has significant advantages over the recently reported FLIM probes,^9^ that is, long lifetimes and high pH-sensitivity. An intracellular pH(±2σ) of 6.97 ± 0.14 was calculated for C3H10T1/2 cells using the probe CdSe/ZnS-*PH*. This value is consistent with the intracellular pH value reported for the same type of cells employing the probe CdSe/ZnS-*P.*^9^ Interestingly, the fluorescence lifetime and the estimated intracellular pH remain nearly constant in C3H10T1/2 cells treated with CdSe/ZnS-*PH* for 24 h. Considering all these results, CdSe/ZnS-*PH* emerges as a promising nanoprobe for fluorescence lifetime bioimaging.

## Supporting Information Available

Additional details of materials and methodologies, additional figures of nanoparticle functionalization, characterization, and pH-sensitivity mechanism (Figures S1–S9 and Table S1), additional details of the fitting of pH-titration curves (Figure S10 and Tables S2 and S3), an additional figure of cytotoxicity assays (Figure S11), and additional FLIM images and fluorescence lifetime histograms (Figures S12–S16) (PDF)
