# Supplementary material for: A Novel Quantum Dot-Based pH Probe for Long-Term Fluorescence Lifetime Imaging Microscopy Experiments in Living Cells
Source: ACS Appl Mater Interfaces. 2022 Jan 10;14(2):2578–86. doi: 10.1021/acsami.1c19926 (PMC8778634; doi:10.1021/acsami.1c19926)
Supplement: Supplementary file 1 — am1c19926_si_001.pdf [file am1c19926_si_001.pdf]

A Novel QD-based pH Probe for Long-term FLIM experiments in Living Cells

Diego Herrera-Ochoa[†], Pedro J. Pacheco-Liñán[†], Iván Bravo[†,§], and Andrés Garzón-Ruiz[†,*]

[†]*Departamento de Química Física, Facultad de Farmacia, Universidad de Castilla-La Mancha, Av. Dr. José María Sánchez Ibáñez, s/n, 02071 Albacete, Spain.*
[§]*Centro Regional de Investigaciones Biomédicas (CRIB), Unidad Asociada de Biomedicina (UCLM-CSIC), C/ Almansa, 14, 02008 Albacete, Spain*

SUPPORTING INFORMATION

Content:

1. Details on Materials and Methodologies	
2. Nanoparticle functionalization, characterization, and pH-sensitivity mechanism	**Figures S1-S9, Table S1**
3. Additional details on the fitting of pH titration curves	**Figure S10, Tables S2 and S3**
4. Cytotoxicity assays	**Figure S11**
5. Additional FLIM images	**Figures S12-S16**
6. References	

1. Details on Materials and Methods

1.1. Materials

CdSe/ZnS core/shell QD (λ_{em} maximum of 620 nm), Ficoll400, bovine serum albumin (BSA), glucose, Tris-HCl buffer, chloroform (spectroscopy grade), nigericin sodium salt, inorganic salts (NaCl, KCl, $MgSO_4$, and $CaCl_2$) and N-acetylcysteine were acquired from Sigma-Aldrich. Thiazolyl blue tetrazolium bromide (MTT) was purchased from Acros. H–D-penicillamine–histidine–OH (*PH*) peptide was synthesized by DestiNA Genomics S.L (Spain). Dulbecco's modified Eagle's medium (DMEM), DMEM without phenol red and fetal bovine serum (FBS) were obtained from Lonza. The pH of the aqueous solutions and buffers was adjusted using NaOH and HCl. The used reagents were of molecular biology grade and the stock solutions were kept at 4°C and in the dark. All the buffer solutions were prepared in Milli Q water.

1.2. Functionalization procedure

a) Functionalization of CdSe/ZnS-*PH*

2 mL of QD in chloroform solution (0.5 mg/mL; ~1.16 µM) were mixed with 0.33 mL of a methanolic solution of the peptide PH (10 mg/mL; 35 mM). Then, 10 µL of NaOH (10 M) were added to the previous solution and sonicated for 15 minutes. Subsequently, 500 µL of PBS (phosphate-buffered solution; pH 7.4) were added and the obtained solution was mixed vigorously. After that, the upper aqueous layer was collected. The previous step was repeated with 300 µL of PBS solution. The collected aqueous solutions were mixed with ethanol (ratio 1:1) and centrifuged at 14.000 rpm for 10 minutes at 4°C. The precipitated nanoparticle was re-suspended in 1 mL of PBS solution [1,2].

b) Functionalization of CdSe/ZnS-*A*

2 mL of QD in chloroform (0.5 mg/mL; ~1.16 µM) were mixed with 35.6 µL of an aqueous solution of N-acetylcysteine (10 mg/mL; 61 mM). Then, 500 µL of ethanol were added to the previous solution and sonicated for 5 minutes. 500 µL of PBS (pH 7.4) were added and the obtained solution was mixed vigorously. After that, the top aqueous layer was collected. The previous step was repeated with 300 µL of PBS solution. The collected aqueous solutions were mixed with ethanol (ratio 1:1) and were centrifuged at

14.000 rpm for 10 minutes at 4°C. The precipitated nanoparticle was re-suspended in 1 mL of PBS solution [1,3].

1.3. Nanoparticle characterization

Hydrodynamic radius, polydispersity (PDI) and zeta potential measurements were acquired using a Zetasizer Nano ZS (Malvern Panalytical) dynamic light scattering equipment. Nanoparticle solutions (0.6 μM) were prepared in Tris-HCl buffer at different pHs (3.0, 5.0, 7.0 and 9.0). For zeta potential determination, each sample was measured three times with 50 scans for each measurement. In addition, hydrodynamic size and PDI were daily measured to monitor the stability of the nanoparticles against aggregation.

Transmission electron microscope (TEM) images of the nanoparticles were acquired on a Jeol JEM 2100 TEM microscope operating at 200 kV and equipped with an Oxford Link EDS detector. The resulting images were analyzed using Digital Micrograph™ software from Gatan.

Ligand grafting densities were estimated by UV-Vis absorption spectroscopic measurements. A calibration line, according to the Lambert-Beer´s law, was performed for each ligand in PBS solution (pH 7.4). These calibration lines were employed to determine the concentration of ligand before and after the functionalization (the quantity of bound ligand to QDs were obtained from these concentrations). The concentration of QD was also determined by UV-Vis absorption spectroscopy (in chloroform, ε_{QD} = 5.6×10^5 M^{-1} cm^{-1} at the first exciton peak; data provided by the supplier, Sigma-Aldrich). The QD surface was calculated considering a diameter of 8.1 nm (obtained by transmission electron microscopy, TEM).

1.4. Absorption and Fluorescence Measurements

UV-Vis absorption spectra were recorded at 298 K on a V-750 spectrophotometer (JASCO) using a slit width of 0.4 nm and a scan rate of 600 nm/min. Steady-state fluorescence (SSF) and time-resolved fluorescence (TRF) spectra were acquired on an FLS920 spectrofluorometer (Edinburgh Instruments) equipped with a MCP-PMT (microchannel plate-photomultiplier tube) detector (R3809 model) and a TCSPC (time-correlated single photon counting) data acquisition card (TCC900 model). The temperature of the sample, fixed at 20ºC, was controlled by a TLC 50 cuvette holder (Quantum Northwest). A Xe lamp (450 W) was used as light source for the acquisition of the SSF spectra. Excitation and emission slits ($\Delta\lambda_{ex}$ and $\Delta\lambda_{em}$, respectively) were both

fixed at 4 nm, the step was 1 nm, and the dwell time was 0.1 s. A sub-nanosecond pulsed Light-Emitting Diode, EPLED-560 (Edinburgh Photonics) was employed as light source at 565 nm for the acquisition of the fluorescence decays (TRF spectra). The excitation wavelength (λ_{ex}), emission wavelength (λ_{em}), and $\Delta\lambda_{em}$ and acquisition time were fixed at 565 nm, 626 nm, 10 nm, and 300 s, respectively. Fluorescence decay profiles were fitted using the F980 software (Edinburgh Instruments). Two different media were employed for the pH-dependence experiments: (i) Tris-HCl buffer solutions (10 mM); (ii) a synthetic intracellular buffer, SIB (10 mM Tris buffer, 1 mM Mg^{2+}, 1 mM Ca^{2+}, 50 mM Na^{+}, 150 mM K^{+}, 1% Ficoll400 and 0.2 mg/mL BSA).

Fluorescence quantum yield measurements were carried out in Tris-HCl buffer solution and sample concentration of 20 nM. The measures were performed in a FS5 spectrofluorometer (Edinburgh Instruments) equipped with an integrating sphere, a 150 Xe lamp as light source, and a PMT (photomultiplier tube) detector (R928P model). The excitation wavelength, excitation and emission slits, step and dwell time were fixed at 511 nm, 10 and 0.15 nm, 1 nm and 0.2 s, respectively.

1.5. Fluorescence Lifetime Imaging of Cells

C3H/10T1/2 cells were seeded onto 20 mm square glass cover slides into 6-well plates and cultured with DMEM medium without phenol red at 37°C in a 5% CO_2 humidified atmosphere until the cells reached 50−80% confluence. The cells were incubated with CdSe/ZnS-*PH* (50 nM) in serum-free DMEM medium without phenol red for 60 min. After incubation, the cells were washed five times with PBS.

After the treatment, FLIM images of the cells were acquired using a MicroTime 200 microscope (PicoQuant) equipped with a TCSPC card and two TAU-SPAD-100 avalanche photodiode detectors. Samples were excited with a 511 nm diode pulse laser with a repetition rate of 10 MHz and ~0.9 μW power excitation. TCSPC resolution was 16 ps and the emission was recorded between 605 to 635 nm with an HC617/14 bandpass filter. 80×80 μm regions were scanned with 156 nm/pixel spatial resolution and a dwell time of 2 ms. FLIM images were processed using SymphoTime64 software (PicoQuant). A pixel binning of 2 points was used for all the images (see reference [1] for more details).

1.6. Intracellular pH Modification

C3H/10T1/2 cells were incubated with CdSe/ZnS-*PH* as explained in the previous section. Afterward, cells were incubated for 10 min at 37 °C with nigericin (10 μM)

solved in an extracellular buffer composed by different proportions of KH_2PO_4/NaCl (135 and 20 mM, respectively) and K_2HPO_4/NaCl (110 mM and 20 mM, respectively) to achieve different pH values. Finally, FLIM images of the treated cells were recorded (see reference [1] for more details).

2. Nanoparticle functionalization, characterization and pH-sensitivity mechanism

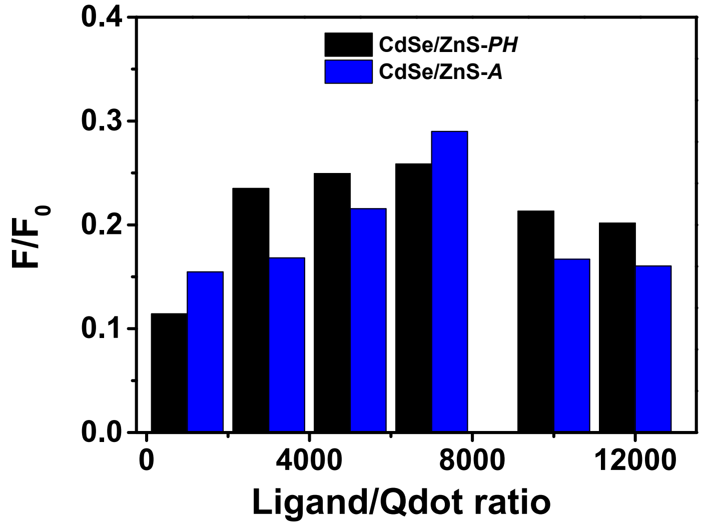

Figure S1. Evolution of the fluorescence emission intensity (F) of the nanoparticles functionalized with different ligand:QD ratios (pH 7.4; QD concentration of 10 nM). F_0 is the fluorescence intensity of a reference sample (CdSe/ZnS 10 nM solved in chloroform) (λ_{ex} = 511 nm; λ_{em} = 626 nm; $\Delta\lambda_{ex}$ = $\Delta\lambda_{em}$ = 2 nm).

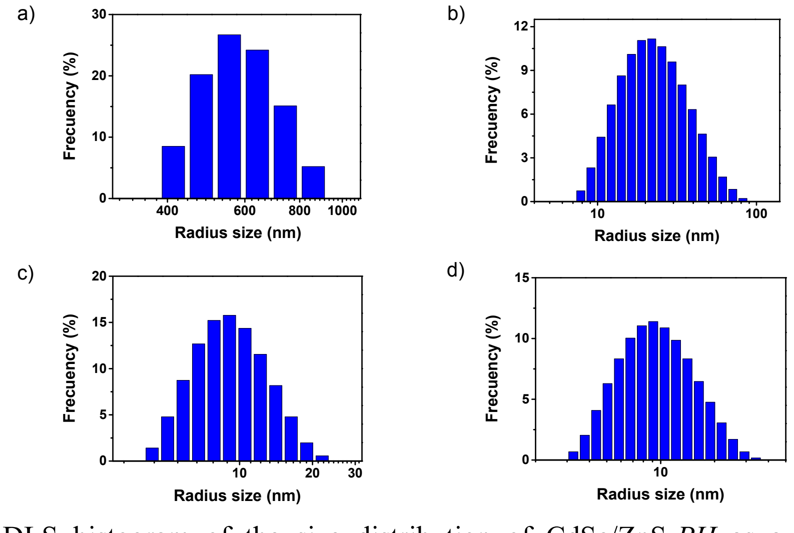

Figure S2. DLS histogram of the size distribution of CdSe/ZnS-*PH* as a function of pH (nanoparticle concentration of 0.6 μM): (a) pH 3.0, (b) pH 5.0, (c) pH 7.0, and (d) pH 9.0

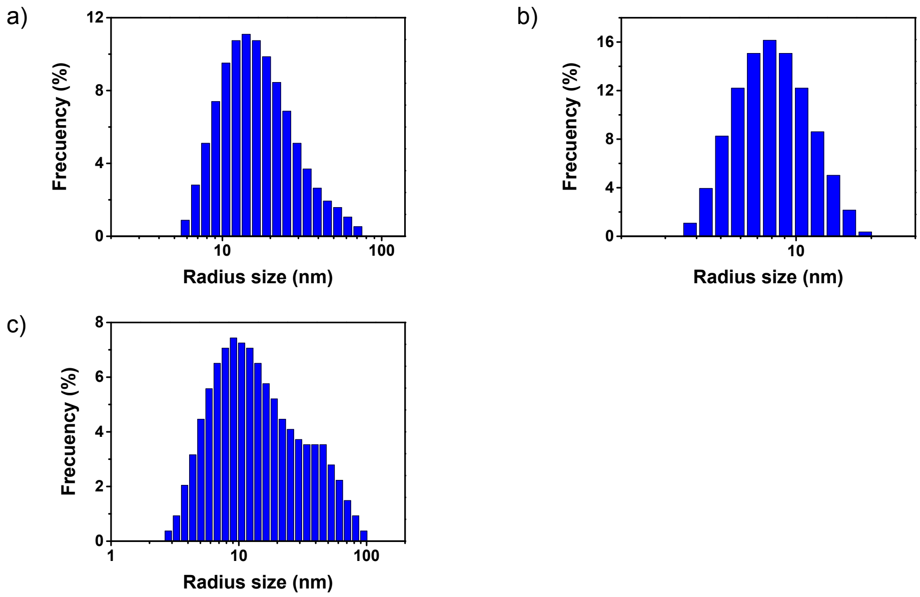

Figure S3. DLS histogram of the size distribution of CdSe/ZnS-*A* as a function of pH (nanoparticle concentration of 0.6 μM): (a) pH 5.0, (b) pH 7.0, and (c) pH 9.0

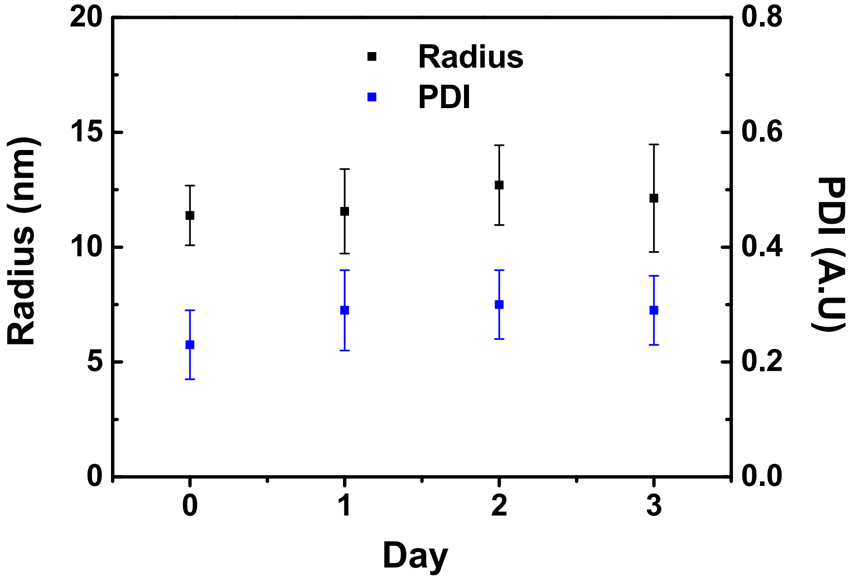

Figure S4. Evolution of the size (radius) of CdSe/ZnS-*PH* as a function of the time. Measures were carried out at pH = 7.4 and nanoparticle concentration of 0.6 μM.

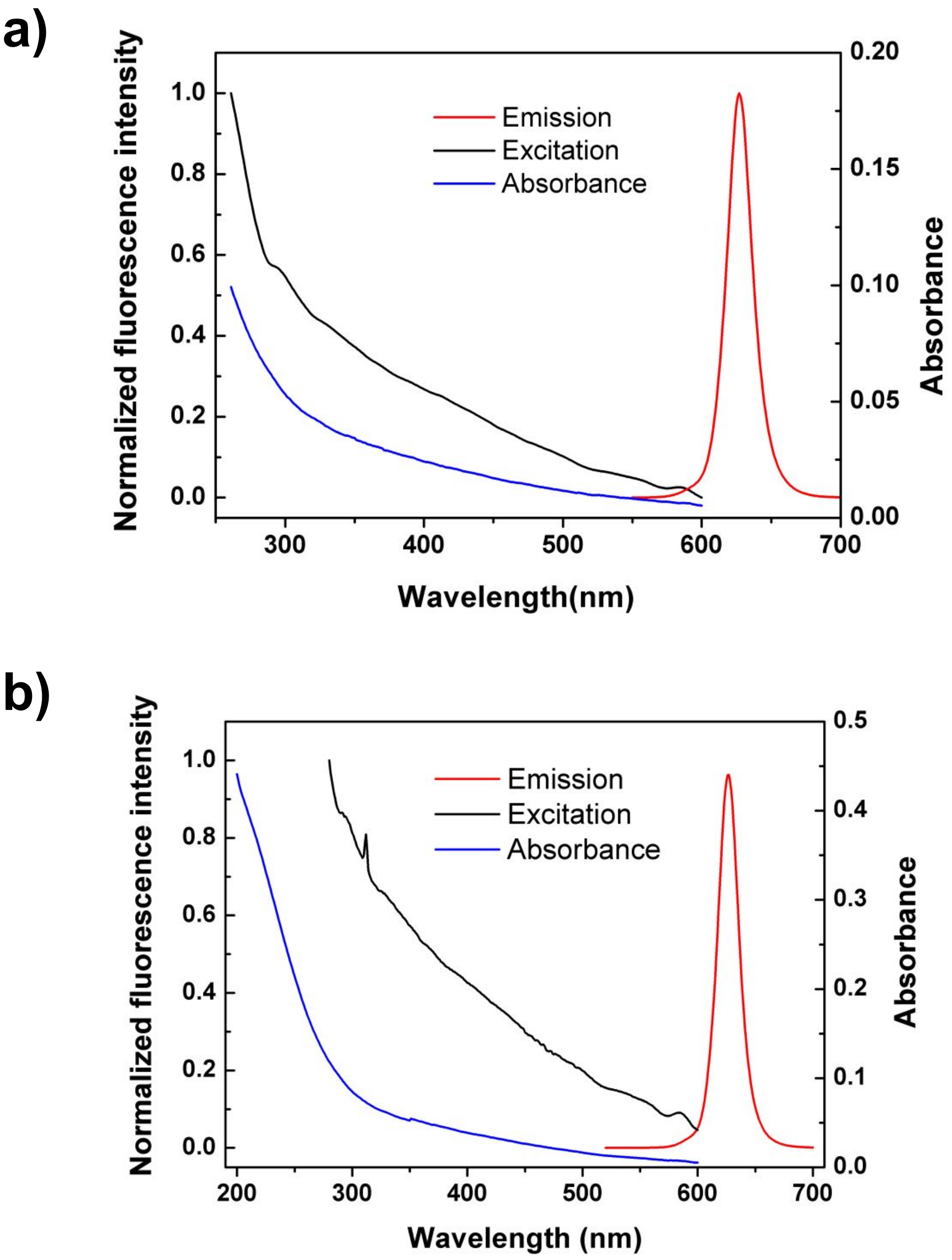

Figure S5. Absorption, excitation and emission spectra of (a) CdSe/ZnS-*PH* and (b) CdSe/ZnS-*A* in aqueous solution (Tris-HCl buffer, pH 7.4, nanoparticle concentration of 10 nM).

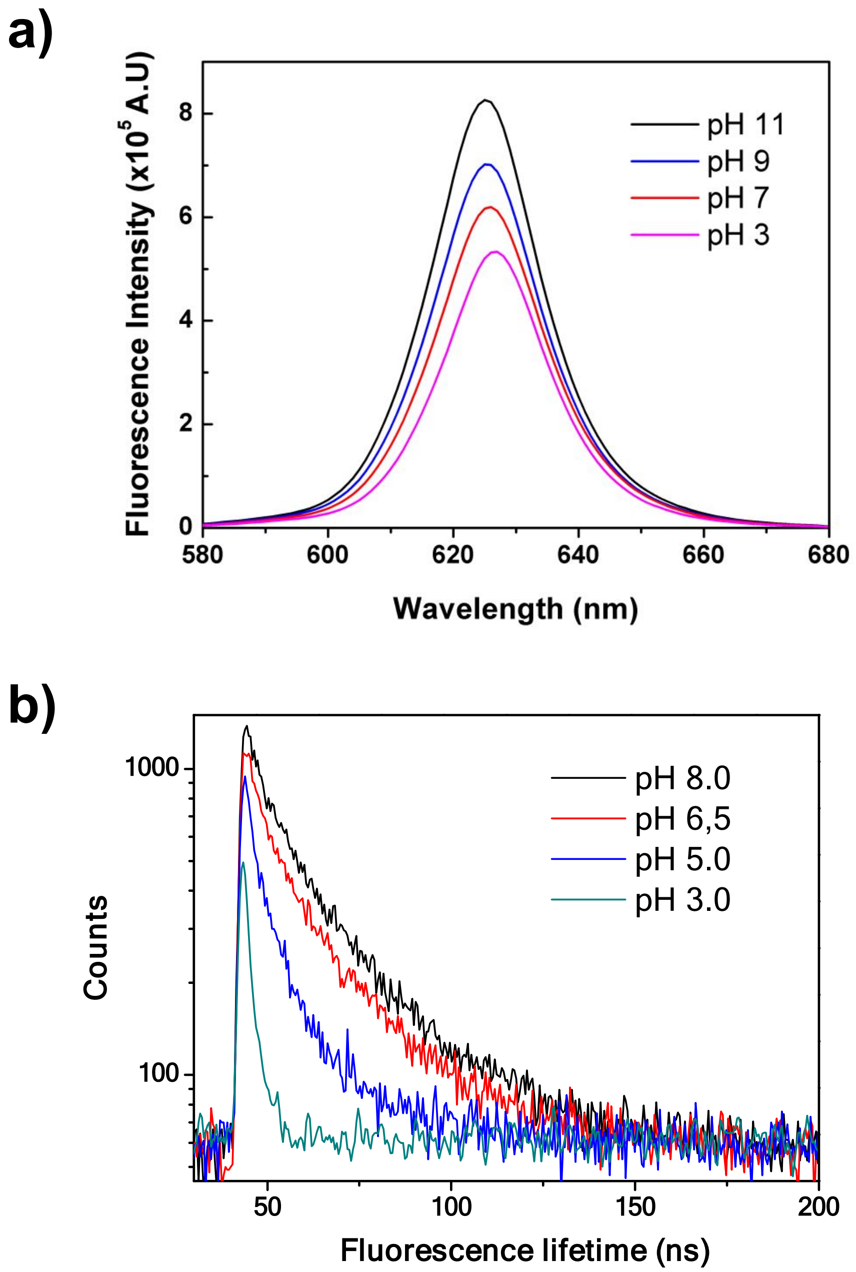

Figure S6. (a) Steady-state and (b) time-resolved fluorescence measurements of CdSe/ZnS-*A* in aqueous solution at different pH values (employing Tris-HCl buffer and nanoparticle concentration of 10 nM).

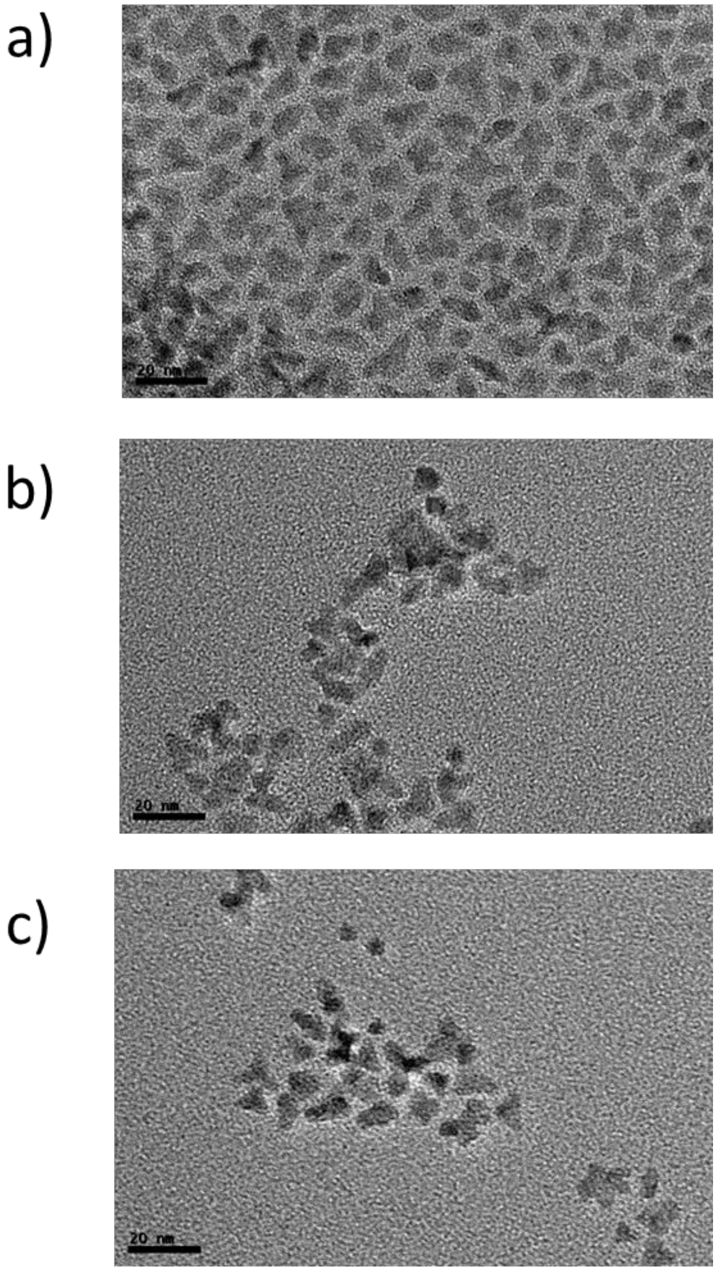

Figure S7. TEM images obtained for non-functionalized CdSe/ZnS quantum dots.

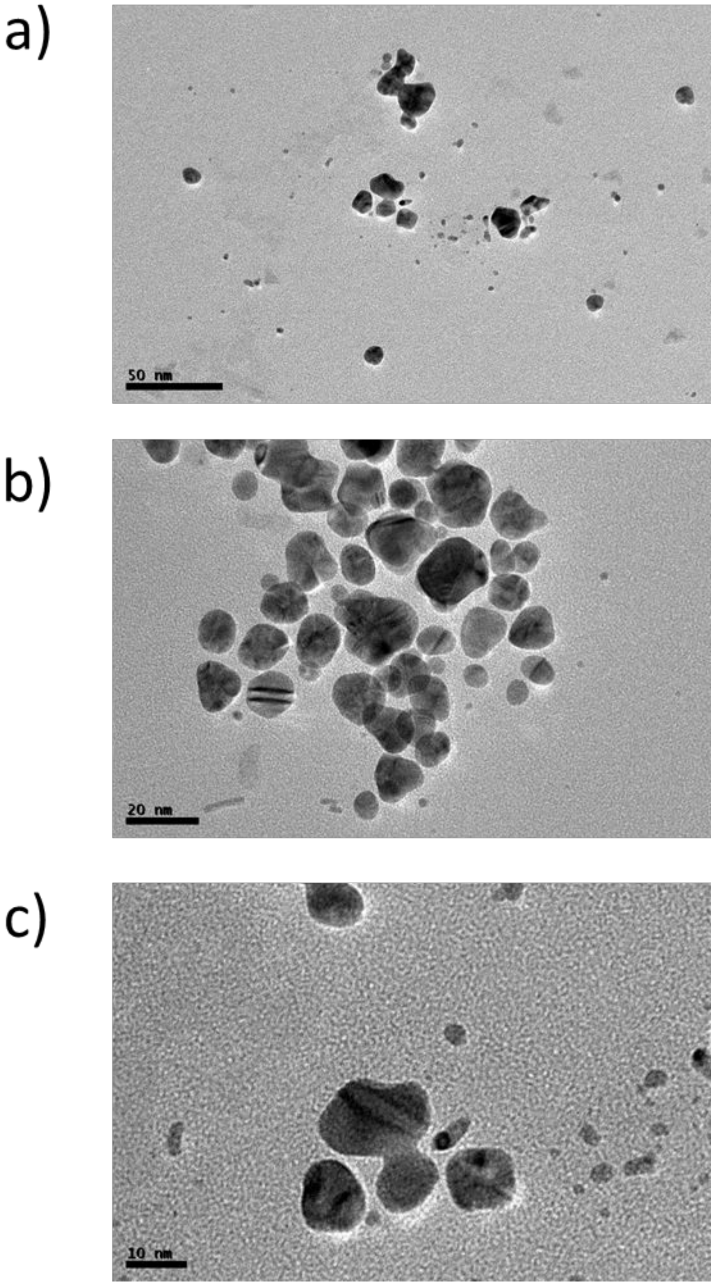

Figure S8. TEM images obtained for CdSe/ZnS-*PH*.

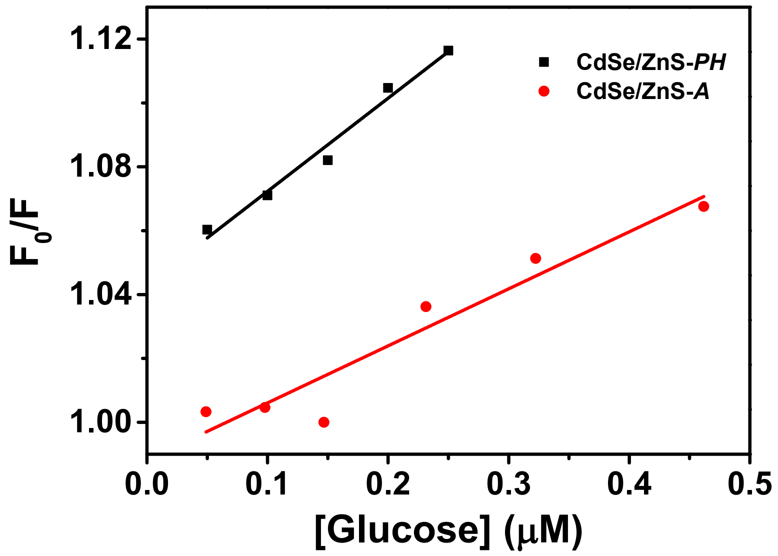

Figure S9. Stern-Volmer plots obtained for CdSe/ZnS-*PH* and the reference nanoparticle (CdSe/ZnS-*P*). F_0 is fluorescence intensity determined for the nanoparticle in absence of quencher. F is the fluorescence intensity recorded for the nanoparticle at different concentrations of quencher (glucose).

Table S1. Ligand:QD ratio and number of ligands per nm^{-2} estimated the functionalized nanoparticles

Compound	Ligand:QD ratio	Ligands nm^{-2} [a]
CdSe/ZnS-*PH*	674	3.3
CdSe/ZnS-*A*	1314	6.4
CdSe/ZnS-*P*	1824	8.9

[a] This calculation was carried out considering a QD surface of 206 nm^2. The QD surface was calculated using a value of 8.1 nm as QD diameter (determined by TEM in this work).

3. Additional details on the fitting of pH titration curves

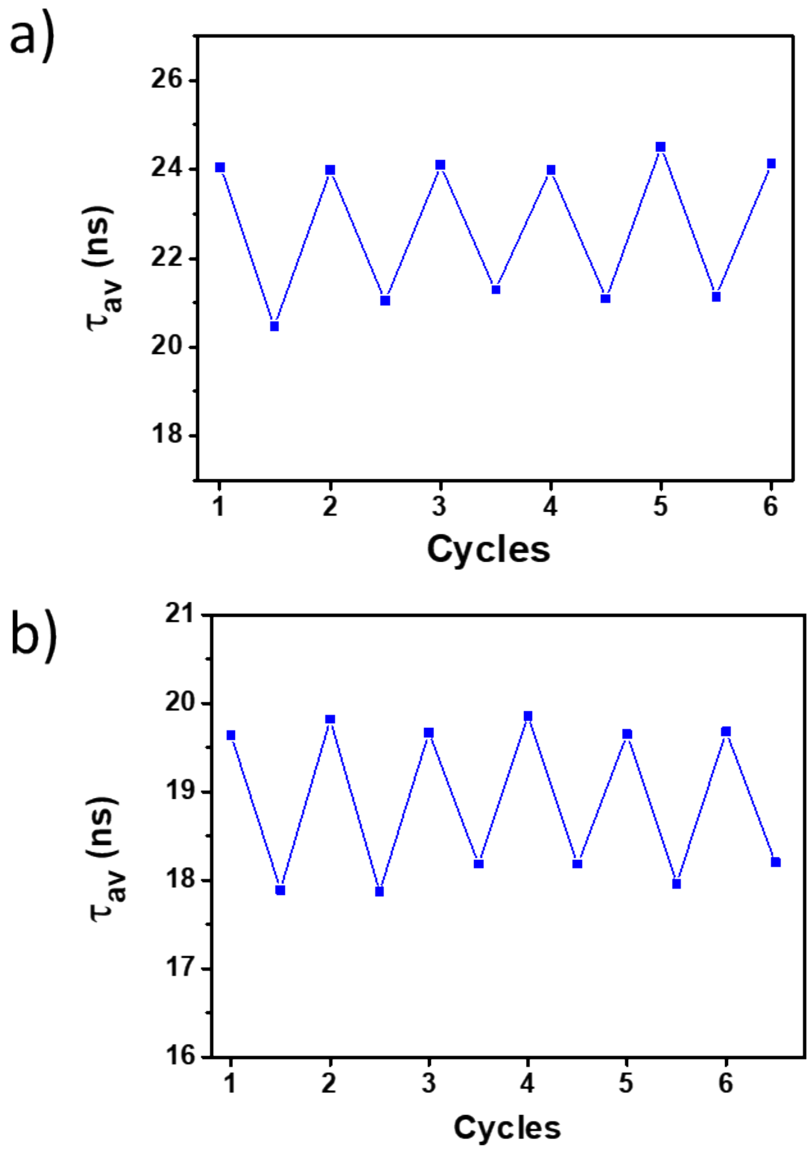

Figure S10. Reversibility of the pH-sensitive response of (a) CdSe/ZnS-*PH* and (b) CdSe/ZnS-*A* (between pH 6.5 and 7.5)

Table S2. Fluorescence lifetimes (along with their standard deviation, ±2σ) determined for CdSe/ZnS-*PH* and CdSe/ZnS-*A* at different pH values (employing Tris-HCl buffer and nanoparticle concentration of 10 nM).

**Nanoparticle**	**pH**	3.0	4.0	5.0	6.0	6.5	7.0	7.5	8.0
CdSe/ZnS-*PH*	**τ_{av} (ns)**	8.0	13.7	16.2	22.4	25.1	26.0	26.8	26.3
	**±2σ**	1.5	0.5	1.3	2.2	1.4	0.9	0.3	0.9
CdSe/ZnS-*A*	**τ_{av} (ns)**	1.9	2.7	11.0	18.0	17.9	19.5	20.0	20.3
	**±2σ**	0.1	0.1	0.2	1.6	2.9	1.4	2.6	2.8

Table S3. Sigmoidal fitting parameters of τ_{av} *vs*. pH corresponding to the titration curves showed in Figure 2c.

**Compound**	**$A_1 \pm 2\sigma$ (ns)**	**$A_2 \pm 2\sigma$ (ns)**	**$pK_a \pm 2\sigma$**	**$p \pm 2\sigma$**	**r^2**
CdSe/ZnS-*PH*	8.35±1.47	26.81±0.53	4.74±0.28	0.56±0.16	0.98
CdSe/ZnS-*A*	1.90±0.05	19.27±0.37	4.97±0.01	1.40±0.04	0.99
CdSe/ZnS-*P* [a]	3.94±0.01	13.75±0.64	5.70±0.11	1.32±0.44	0.98

[a] Data extracted from reference [1] for comparative porpoises

4. Cytotoxicity assays

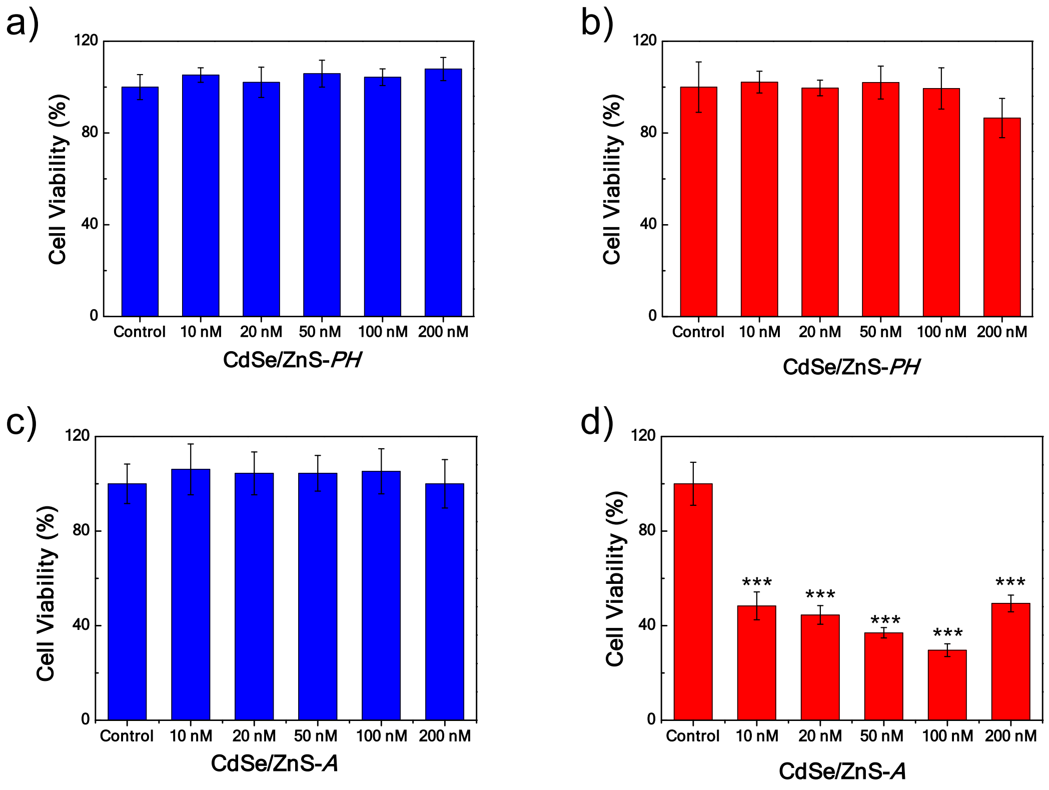

Figure S11. Cytotoxic effect of (a, b) CdSe/ZnS-*PH*, (c, d) CdSe/ZnS-*A* from 10 to 200 nM concentration in C3H10T1/2 cells treated for (a, c) 2 h and (b, d) 24 h at 37 ºC. Control sample correspond to cells not treated with nanoparticles (** $p < 0.01$ and *** $p < 0.001$ compared to control).

5. Additional FLIM measurements

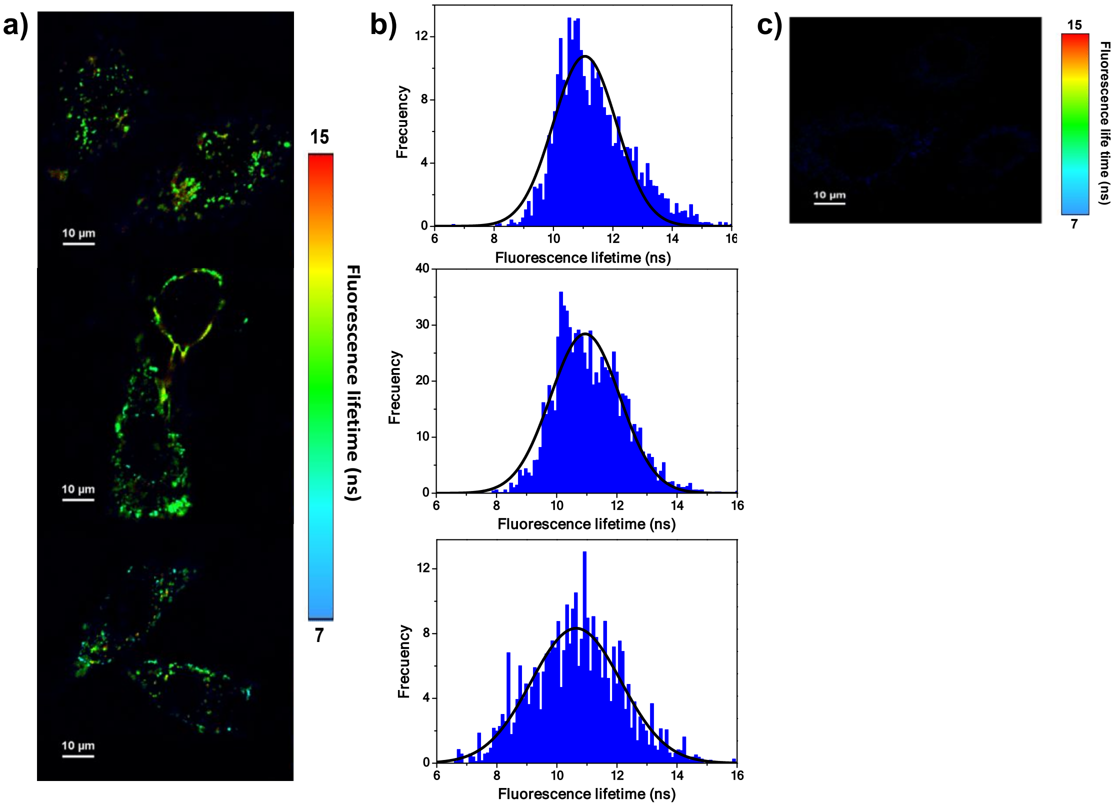

Figure S12. (a) Examples of FLIM images aquired for C3H10T1/2 cells treated with CdSe/ZnS-*PH* (50 nM) for 30 min. (b) Fluorescence lifetime histograms collected from FLIM image. (c) FLIM images aquiered for control cells.

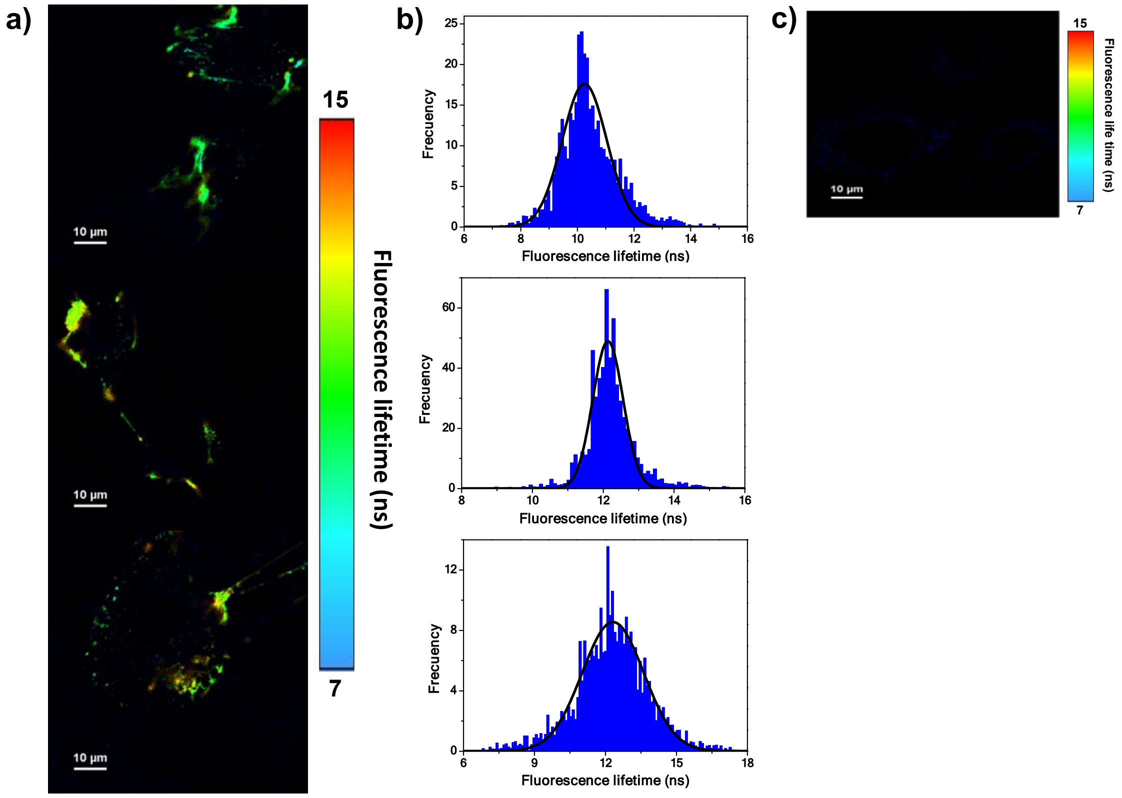

Figure S13. (a) Examples of FLIM images aquired for C3H10T1/2 cells treated with CdSe/ZnS-*A* (50 nM) for 30 min. (b) Fluorescence lifetime histograms collected from FLIM images. (c) FLIM image aquiered for control cells.

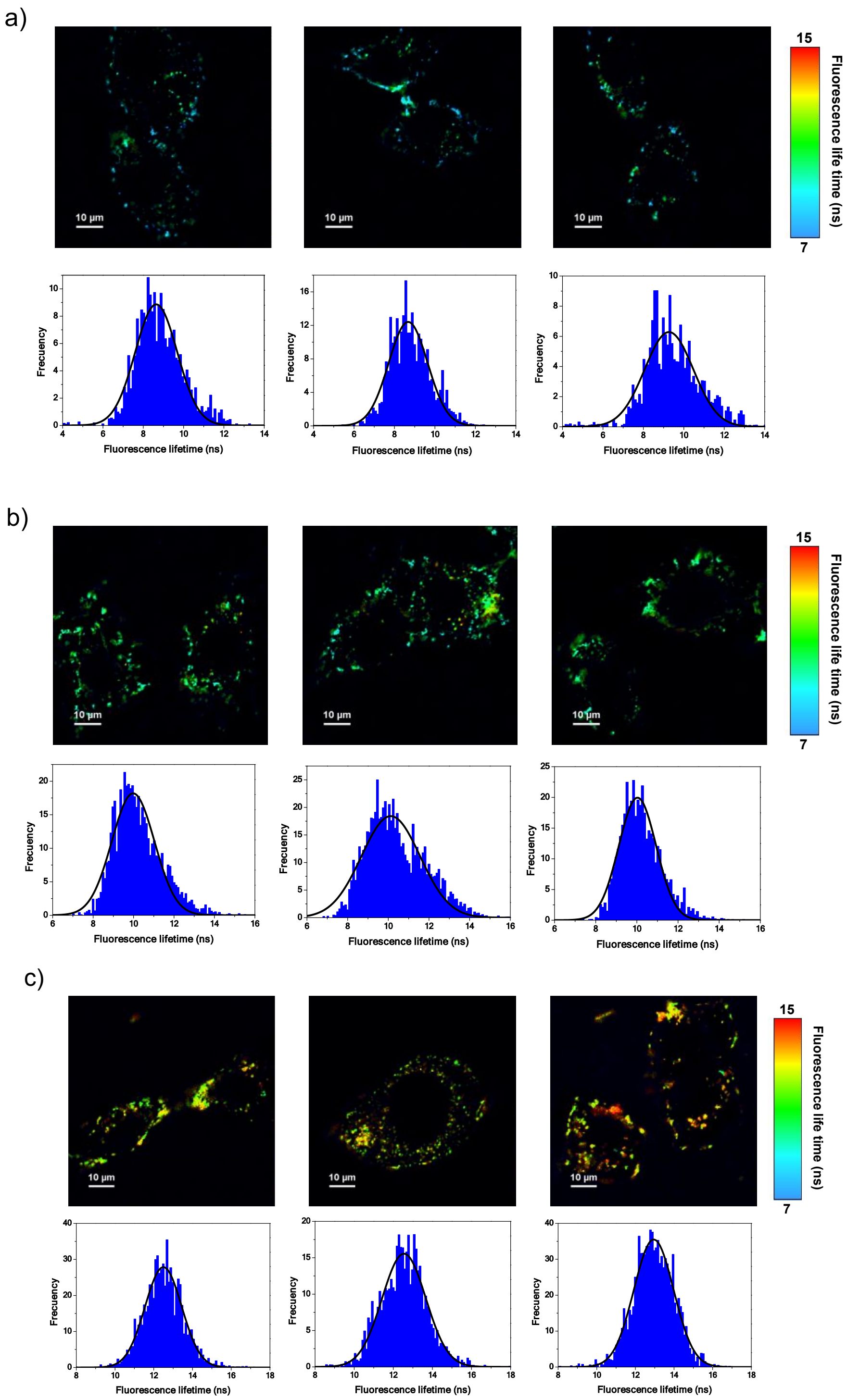

Figure S14. pH sensitivity experiment: examples of FLIM images, along with their respecive fluorescence lifetime histograms, aquired for C3H10T1/2 cells treated with CdSe/ZnS-*PH* (50 nM) for 30 minutes whose intracellular pH was artificially modified: (a) pH = 6.4; (b) pH = 6.8; (c) pH = 7.2.

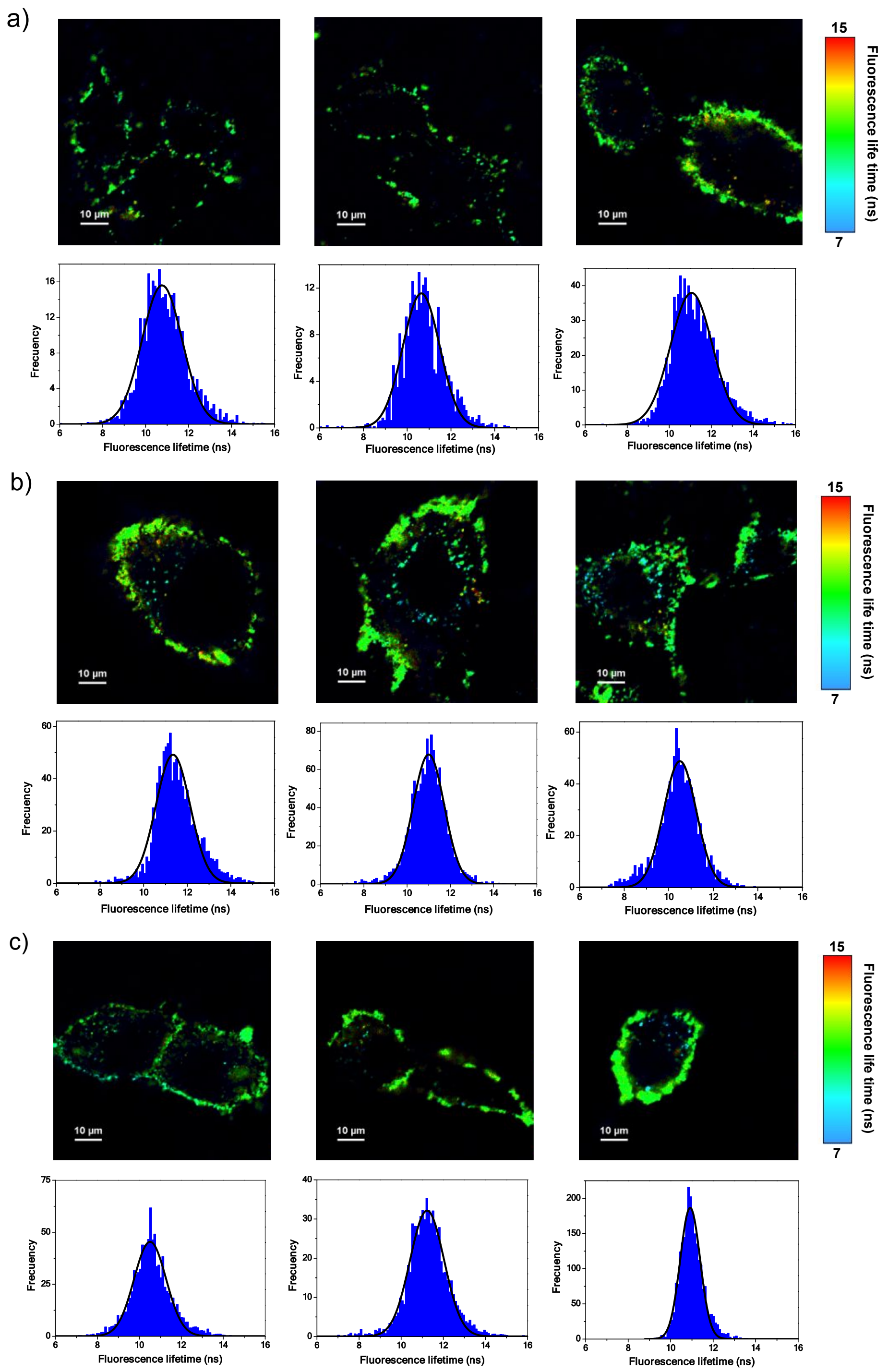

Figure S15. Temporal stability experiment: examples of FLIM images, along with their respecive fluorescence lifetime histograms, aquired for C3H10T1/2 cells after (a) 2h, (b) 6h and (c) 24h of treatment with CdSe/ZnS-*PH* (50 nM). In this experiment, pH was not artificially modified.

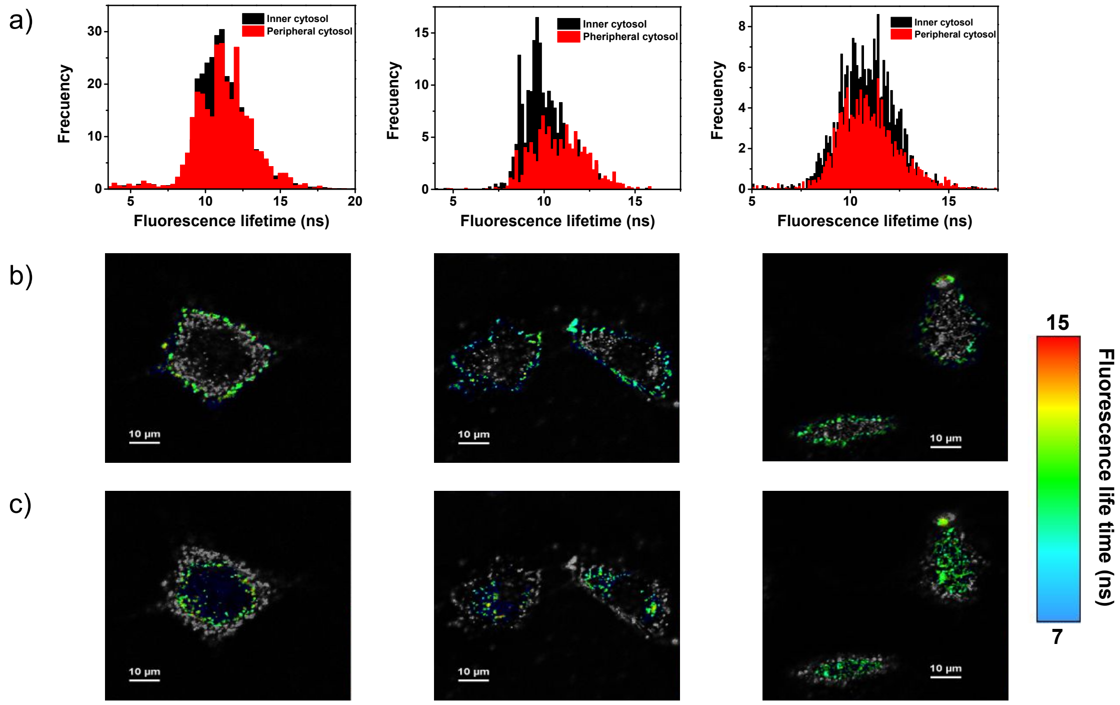

Figure S16. (a) Fluorescence lifetime histograms obtained for two ROIs (regions of interest) of FLIM images. (b) FLIM image of ROI-1: peripheral regions of cytosol. (c) FLIM image of ROI-2: inner cytosol.

6. References

[1] Pacheco-Liñán, P.J.; Bravo, I.; Nueda, M.L.; Albaladejo, J.; Garzón-Ruiz, A. Functionalized CdSe/ZnS Quantum Dots for Intracellular pH Measurements by Fluorescence Lifetime Imaging Microscopy. *ACS Sens*. **2020**, *5*, 2106−2117.

[2] Pratiwi, F.W.; Hsia, C.-H.; Kuo, C.W.; Yang, S.-M.; Hwu, Y.-K.; Chen, P. Construction of Single Fluorophore Ratiometric pH Sensors Using Dual-Emission Mn^{2+}-Doped Quantum Dots. Biosens. Bioelectron. **2016**, *84*, 133–140.

[3] Chen, G.; Zhang, Y.; Peng, Z.; Huang, D.; Li, C.; Wang, Q. Glutathione-Capped Quantum Dots for Plasma Membrane Labeling and Membrane Potential Imaging. Nano Res. 2019, 12 (6), 1321–1326.
